# GeneAI 3.0: powerful, novel, generalized hybrid and ensemble deep learning frameworks for miRNA species classification of stationary patterns from nucleotides

**DOI:** 10.1038/s41598-024-56786-9

**Published:** 2024-03-26

**Authors:** Jaskaran Singh, Narendra N. Khanna, Ranjeet K. Rout, Narpinder Singh, John R. Laird, Inder M. Singh, Mannudeep K. Kalra, Laura E. Mantella, Amer M. Johri, Esma R. Isenovic, Mostafa M. Fouda, Luca Saba, Mostafa Fatemi, Jasjit S. Suri

**Affiliations:** 1 Department of Computer Science, Graphic Era Deemed to be University, Dehradun, Uttarakhand India; 2 Department of Cardiology, Indraprastha APOLLO Hospitals, New Delhi, India; 3 Department of Computer Science and Engineering, NIT Srinagar, Hazratbal, Srinagar, India; 4 Department of Food Science, Graphic Era Deemed to be University, Dehradun, Uttarakhand India; 5 Heart and Vascular Institute, Adventist Health St. Helena, St Helena, CA USA; 6 Advanced Cardiac and Vascular Institute, Sacramento, CA USA; 7 Department of Radiology, Massachusetts General Hospital, Boston, MA 02115 USA; 8 Department of Biomedical and Molecular Sciences, Queen’s University, Kingston, ON Canada; 9 Laboratory for Molecular Genetics and Radiobiology, University of Belgrade, Belgrade, Serbia; 10 Department of Electrical and Computer Engineering, Idaho State University, Pocatello, ID 83209 USA; 11 Department of Neurology, University of Cagliari, Cagliari, Italy; 12 Department of Physiology and Biomedical Engineering, Mayo Clinic, Rochester, MN 55905 USA; 13 Stroke Monitoring and Diagnostic Division, AtheroPoint LLC, Roseville, CA 95661 USA

**Keywords:** miRNA classification, Ensemble deep learning, Ensemble machine learning, Composite feature extraction, Validation, Statistical tests, Computer science, Software, Statistics, Genetics, Genomics

## Abstract

Due to the intricate relationship between the small non-coding ribonucleic acid (miRNA) sequences, the classification of miRNA species, namely Human, Gorilla, Rat, and Mouse is challenging. Previous methods are not robust and accurate. In this study, we present AtheroPoint’s GeneAI 3.0, a powerful, novel, and generalized method for extracting features from the fixed patterns of purines and pyrimidines in each miRNA sequence in ensemble paradigms in machine learning (EML) and convolutional neural network (CNN)-based deep learning (EDL) frameworks. GeneAI 3.0 utilized five *conventional* (Entropy, Dissimilarity, Energy, Homogeneity, and Contrast), and three *contemporary* (Shannon entropy, Hurst exponent, Fractal dimension) features, to generate a *composite* feature set from given miRNA sequences which were then passed into our ML and DL classification framework. A set of 11 new classifiers was designed consisting of 5 EML and 6 EDL for binary/multiclass classification. It was benchmarked against 9 solo ML (SML), 6 solo DL (SDL), 12 hybrid DL (HDL) models, resulting in a total of 11 + 27 = 38 models were designed. Four hypotheses were formulated and validated using explainable AI (XAI) as well as reliability/statistical tests. The order of the mean performance using accuracy (ACC)/area-under-the-curve (AUC) of the 24 DL classifiers was: EDL > HDL > SDL. The mean performance of EDL models with CNN layers was superior to that without CNN layers by 0.73%/0.92%. Mean performance of EML models was superior to SML models with improvements of ACC/AUC by 6.24%/6.46%. EDL models performed significantly better than EML models, with a mean increase in ACC/AUC of 7.09%/6.96%. The GeneAI 3.0 tool produced expected XAI feature plots, and the statistical tests showed significant *p*-values. Ensemble models with composite features are highly effective and generalized models for effectively classifying miRNA sequences.

## Introduction

MicroRNAs (miRNAs) are short RNA molecules that play a crucial role in regulating gene expression^1,2^. Typically consisting of 20–25 nucleotides, they are formed through the transcription of longer RNA molecules by cellular enzymes. By binding to target messenger RNA (mRNA), miRNAs can inhibit mRNA’s translation, thereby controlling the expression of specific genes. This mechanism influences various biological processes such as proliferation^3^, apoptosis^4^, development^5,6^, and differentiation^7^. Disruptions in miRNA expression have been associated with diseases like cancer^8–10^ and cardiovascular disease^11–13^. Accurately classifying miRNA sequences based on their origin^14–16^ is crucial due to the diverse roles that miRNA sequences play in disease development across different species^17–19^. This classification enables the identification of conserved miRNA sequences and their target genes, contributing to a better understanding of miRNA function and the detection of potential threats^20–22^.

Machine learning’s application has been constantly observed in multiple bioinformatics studies^23–33^, including several tools have gained attention in the field of miRNA identification. These tools include Mipred^25^, Triplet^34^, HeteroMirPred^35^, micropred^36^, PlantMiRNAPred^37^, and mirnaDetect^38^. They have the ability to extract pre-miRNAs from protein-coding regions that exhibit stem-loop structures similar to genuine pre-miRNAs but have not been identified as such. In addition, numerous computational methods have been developed to enhance miRNA identification. These methods include MatureByes^39^, MiRMat^40^, MiRRim2^41^, MiRdup^42^, MaturePred^43^, MiRPara^44^, mirExplorer^45^, Matpred^46^, and MiRduplexSVM^47^. MiRNA identification can be performed using de novo methods, which involve computational tools, or by utilizing next-generation sequencing data^48,49^. These methods focus on identifying pre-miRNA sequences that exhibit hairpin-like structures in the input data. They are categorized based on expression-based features or computed sequences.

The intricate nonlinear nature of miRNA sequences poses challenges for these methods, primary due to the high-dimensional feature spaces associated with the sequences^50^. To address these challenges, primitive methods like ensemble ML (EML) methods that employ voting mechanisms^51–53^ have been introduced. This was follwed by deep learning (DL) models, such as Convolutional Neural Networks (CNN) and Long Short-Term Memory (LSTM)^54,55^. DL models have the capability to capture the nonlinear complexity of miRNA sequences, making them well-suited for characterization and classification tasks^54–57^. Despite the promising results achieved by solo DL (SDL) models in miRNA classification, they often require large labeled datasets and are susceptible to overfitting^58^, which hinders their generalization capabilities^59^. To further improve classification performance, hybrid DL (HDL) and ensemble DL (EDL) models have been proposed^60,61^. These models leverage the strengths of multiple DL architectures^62–64^.

Extracting additional features from miRNA sequences is a valuable strategy for overcoming the aforementioned limitations. Although features like k-mer frequency and dinucleotide composition effectively capture sequence-specific details^65–67^, they have inherent limitations in extracting comprehensive information. To address these challenges, conventional features such as Energy, Contrast, and Entropy can be employed to capture structural characteristics^68–71^. Additionally, contemporary features like Shannon Entropy and Hurst Exponent can be derived to obtain additional insights. By combining both sequence-specific and structural features into a composite feature set, the effectiveness of DL models can be further enhanced, resulting in a more robust approach. Further, incorporation of CNN layers in this paradigm enhances classification by capturing local patterns and spatial dependencies. Hence usage of CNN-based EDL models with extracted composite features is paramount in building a robust and state-of-the-art framework for miRNA classification.

In the spirit of improving species classification by employing EDL and EML classifiers, along with novel composite feature extraction we built an extensive set of ensemble-based AI classifiers, focusing on four main hypotheses. First, we investigate the benefits of using EML models with voting compared to SML models for miRNA species classification in binary classification (BC) and multiclass classification (MCC) scenarios. Second, we validate the superiority of EDL models over HDL and SDL models. Additionally, we explore the advantages of incorporating CNN layers in miRNA species classification, comparing them to models *without* CNN layers. Lastly, we examine the advantage of transitioning from EDL models to EML models in ensemble-based species classification. By introducing composite features and enhancing ensemble learning, our approach brings a fresh perspective to design and improves the reliability of genomic sequence testing. Consequently, it enhances the accuracy of miRNA sequence classification, surpassing previous research that relied solely on statistical techniques.

**Figure 1:**
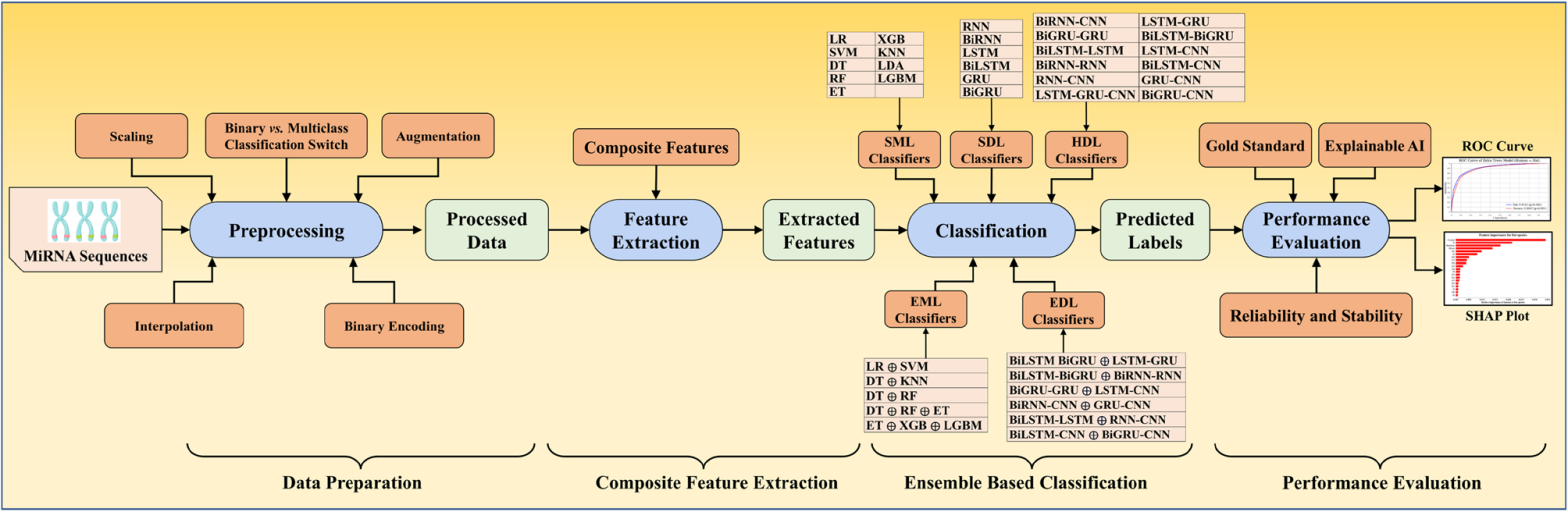
Global architecture of GenAI 3.0 (AtheroPoint LLC, CA, USA). SML: Solo machine learning; EML: Ensemble machine learning; SDL: Solo deep learning; HDL: Hybrid deep learning; EDL: Ensemble deep learning.

Figure 1 presents an overall block diagram of GeneAI 3.0 (AtheroPoint LLC, Roseville, CA, USA). With the input of miRNA species data containing gene sequences, the system performs an intensive data preparation (elliptical preprocessing block), which includes binary encoding of the gene sequence, scaling, augmentation using Adaptive Synthetic Sampling Approach for Imbalanced Learning (ADASYN)^72^, and interpolation. It then performs an elaborated feature extraction (elliptical feature extraction block), where it derives composite features from the binary miRNA sequence. GenAI 3.0 then incorporates 38 extensive AI models (classification block): *nine* SML, *five* EML, *six* SDL, *twelve* HDL and *six* EDL models, and classifies the species along with performance metrics (performance block), consisting of statistical tests and explainable AI (XAI) graphs.

Our research findings validated the advantages of utilizing EDL models in gene classification by conducting experiments that establish the model order as EDL > HDL > SDL. We have also validated the benefits of EML models over SML models in the miRNA classification. Furthermore, we have assessed the performance improvements achieved using EDL models compared to EML models, as well as the advantages gained from incorporating CNN layers in DL models. Alongside our primary contributions, we have investigated the impact of training data size on the model's performance and validated the reliability and stability of our approach through statistical tests. Finally, we have employed XAI plots to interpret our classification findings and offer insights into species classification.

The paper starts elaboration on the methodology which discusses the extracted features, employed classifiers and optimization parameters along with experimental protocols in “Methodology” section. The results are presented in “Results” section, while “Performance evaluation” section provides a performance evaluation with Receiver Operating Characteristic (ROC) curves, and influence of training data size. “Reliability analysis using statistical tests” section demonstrates reliability using statistical tests, while “Explainable artificial intelligence” section uses XAI plots used to enhance the interpretability. “Discussion” section presents a discussion of the principal findings, a benchmarking with previous studies, and an overview of the study's strengths, weaknesses, and extensions. Finally, “Conclusion” section concludes the paper.

## Methodology

In order to explore the connection between miRNA sequences and their corresponding species, we employed statistical ML and DL models for classification in our methodology. The initial stage involved collecting the primary dataset that would serve as the foundation for classification, ensuring its suitability for utilization in the classifiers. Next, we conducted quality control procedures, including categorical encoding of the miRNA sequences, data scaling, oversampling of the minority class, interpolation of missing sequences, and label encoding of the class labels. We also computed both conventional and contemporary features from the dataset. Subsequently, we meticulously designed the architecture of all the AI models used, along with the hyperparameter tuning approaches, loss functions, and training details employed to train these models. Lastly, we defined the performance metrics and experimental protocols utilized in our study.

### Data and data preparation

This study utilized the miRNA Database available at http://www.mirbase.org/ for experimental design, data collection, and discussion purposes. The database encompasses miRNA sequences from various species, including Humans, Gorillas, Mouse, and Rat. Th dataset used in this study consisted of 2654 Human, 369 Gorilla, 1978 Mouse, and 764 Rat miRNA sequences. A ribonucleic acid (RNA) molecule is composed of a backbone comprising sugar ribose and phosphate groups. In contrast to deoxyribonucleic acid (DNA), the sugar ribose lacks deoxyribose and is connected to one of four bases: adenine (A), uracil (U), cytosine (C), or guanine (G). To convert miRNA sequences containing these four bases into binary sequences, we applied a set of rules that mapped each base to a corresponding binary digit^73,74^:

1 $${\text{A}}/{\text{G }} \to { 1} \;{\text{and}}\; {\text{C}}/{\text{U }} \to \, 0$$

This was done using truncation of sequence^75–77^. This resulted in four datasets of binary sequences from the four species: Humans, Gorillas, Mouse, and Rat. Table 1 lists the specifications of each dataset.

**Table 1:** Specifications of the miRNA dataset.

Dataset name	Dataset size
Human	2654
Gorilla	369
Mouse	1978
Rat	764
Combined classes	5765

The class labels for the four species (Human, Gorilla, Mouse, and Rat) were encoded between 0 and 3 to be used as target classes in the classifiers. This label encoding was employed to convert the category labels for each species into numerical values, allowing for the application of DL techniques to analyze the relationships between miRNA sequences and the different species.

To facilitate this analysis, six binary class datasets and four multiclass datasets were prepared. These datasets were carefully curated and preprocessed to cover various scenarios among the four species. In the binary class datasets, two species were compared using a binary classification approach. The *objective* was to accurately differentiate between the two species using the provided dataset features. We created multiple datasets with the aim of achieving generalization^78–81^ in species classification. The purpose behind this initiative was to train our model on a variety of datasets, ensuring its effectiveness in real-life scenarios. This approach allows any gene sequence to be pre-processed, features extracted, and utilized by our model. The binary datasets consisted of the following pairwise species comparisons: Human *vs*. Gorilla, Human *vs*. Rat, Human *vs*. Mouse, Mouse *vs*. Gorilla, Mouse *vs*. Rat, and Gorilla *vs*. Rat. For the multiclass datasets, the methodology used was "one *vs*. all." Each species was considered as one class, while the other three species were treated as the second class. The four multiclass datasets created were: Human *vs*. All, Rat *vs*. All, Gorilla *vs*. All, and Mouse *vs*. All. By utilizing these processed datasets, researchers could leverage DL techniques to gain insights into the relationships between miRNA sequences and different species.

### Data availability/availability of data and materials

Due to its propriety nature, supporting data cannot be made available openly but are available from the corresponding author on reasonable request.

### Quality Control

There is an unbalanced distribution of data points among the various classes in the dataset we acquired for our study. Data size in particular plays a vital role both in generalization vs. memorization protocols. When data size is low, we have seen studies where two types of data augmentation have been adopted^74,79,82–87^. If it is an image data, the data augmentation consisted of increasing the data size by flipping and rotating the images^82–87^. On the other hand, if the data is a point or tabular data, then the augmentation can be accomplished using SMOTE^74^ or ADASYN protocols^88^. To address this issue, we utilized the ADASYN technique, as depicted in Fig. 1. ADASYN is a method that generates synthetic samples for the minority class, thereby achieving a more balanced distribution of data points among the different classes. This approach is beneficial because imbalanced data can hinder the performance of supervised ML algorithms, which often prioritize the majority class and may exhibit poor performance on the minority classes. By employing ADASYN and balancing the representation of the classes in the dataset, we can enhance the performance of various classifiers. Some examples of classifiers that can benefit from this balanced data include Gradient Descent Boosting^89^, Support Vector Machine (SVM)^77^, and Logistic Regression (LR)^88^.

We also employed linear interpolation to handle missing values within the "Human" class. Linear interpolation is a method that estimates the missing values by assuming a linear relationship between the available data points. By applying linear interpolation to the four instances with missing values, we successfully completed the dataset and ensured the integrity of the data for further analysis.

Additionally, to improve the performance of our algorithms on the imbalanced dataset, we implemented data scaling techniques^73,74^ to standardize the features and ensure their similarity in scale. This enabled faster convergence of the algorithms and enhanced the accuracy of predictions. We specifically employed the Min–Max Scaler method, which rescales the data to a fixed range between 0 and 1. This is achieved by subtracting the minimum value and dividing by the range^73,74^. By utilizing this method, we standardized the features and reduced their values, which expedited the training process for both ML and DL models.

### Feature representation and composite features extraction

The miRNA sequence **S**_**t**_ consists of four nucleotide bases: A, C, U, and G, which can be arranged in different combinations. The presence of these nucleotides in the miRNA sequence signifies their interdependencies, and through the analysis of their patterns, distinct characteristics can be identified to distinguish between various species. In order to differentiate species based on feature representations of miRNA sequences, we developed an innovative approach to uncover these nucleotide co-occurrences. To demonstrate the possible arrangements of these nucleotides in miRNA gene sequences, we utilized co-occurrence matrices generated through vector combinations, as depicted in the provided Table 2.

**Table 2:** Possible sets of occurrences of nucleobases A, C, U, and G in an RNA sequence formed by the combination of vectors, where **I, J, K, L, M, N, O,** and** P** are the co-occurrence matrices.

**X**	**Y**	**X**^T^*Y
**X**_1_ = (A, C, U, G)	(A, C, U, G)	**I**_4x4_ = (**X**_1_^T^)_4x1_ x (**Y**)_1x4_
**X**_2_ = (AA, CC, UU, GG)	(A, C, U, G)	**J**_4x4_ = (**X**_2_^T^)_4x1_ x (**Y**)_1x4_
**X**_3_ = (AC, AU, AG, CU, CG, UG)	(A, C, U, G)	**K**_6x4_ = (**X**_3_^T^)_6x1_ x (**Y**)_1x4_
**X**_4_ = (CA, UA, GA, UC, GC, GU)	(A, C, U, G)	**L**_6x4_ = (**X**_4_^T^)_6x1_ x (**Y**)_1x4_
**X**_5_ = (ACU, ACG, AUG, CUG)	(A, C, U, G)	**M**_4x4_ = (**X**_5_^T^)_4x1_ x (**Y**)_1x4_
**X**_6_ = (CAU, CAG, UAG, UCG)	(A, C, U, G)	**N**_4x4_ = (**X**_6_^T^)_4x1_ x (**Y**)_1x4_
**X**_7_ = (AUC, AGC, AGU, CGU)	(A, C, U, G)	**O**_4x4_ = (**X**_7_^T^)_4x1_ x (**Y**)_1x4_
**X**_8_ = (UCA, GCA, GUA, GUC)	(A, C, U, G)	**P**_4x4_ = (**X**_8_^T^)_4x1_ x (**Y**)_1x4_

In order to gain insights into the inherent patterns of miRNA, it is essential to investigate the co-occurrences of nucleobases and analyze both their stationary and non-stationary patterns. To extract valuable information from these patterns, we employed the widely utilized grey-level co-occurrence matrix^90^, a technique commonly employed in texture analysis and pattern recognition^91^. We have adopted the same feature extraction namely entropy, contrast, energy, homogeneity, dissimilarity as previously published by our group^92–96^ Such algorithms are being used for tissue characterization in medical imaging^97,98^. For each miRNA sequence, we computed multiple co-occurrence matrices, namely **I, J, K, L, M, N, O,** and** P**. These matrices captured diverse patterns formed by the nucleobases A, C, U, and G. In Tables (ST1–ST8), we present these co-occurrence matrices, which offer an overview of the different nucleobase arrangements and their corresponding frequencies.

The *primary objective* of constructing co-occurrence matrices from the miRNA sequence **S**_**t**_ is to analyze the occurrence frequency of specific combinations and offsets of the nucleobases A, C, U, and G. The co-occurrence matrix ${\varvec{XC}}{ }$ has a size of $q$ × 4 for a given offset, where $q$ represents the number of distinct nucleobase combinations found in sequence **S**_**t**_. Each element in the co-occurrence matrices presented in Tables (ST1–ST8), denoted as the ($l$, $m$)^th^ position, indicates the frequency of the $l$^*th*^ and $m$^th^ nucleobases occurring in the sequence **S**_**t**_, which has a length of $n$. This relationship can be mathematically expressed using the following equation:

2 $${\varvec{XC}}{ } = { }\mathop \sum \limits_{i = 1}^{n} \mathop \sum \limits_{j = 1}^{n} \left\{ {\begin{array}{*{20}l} {1,} \hfill & {XC \left( {i,j} \right) = l \Lambda XC \left( {i + \Delta i,j + \Delta j} \right) = {\text{ m}}} \hfill \\ {0,} \hfill & {otherwise} \hfill \\ \end{array} } \right.$$

The computation of matrix ${\varvec{XC}}$ is contingent upon the spatial relationship defined by the offset ($\Delta i$, $\Delta j$). These co-occurrence matrices are utilized to analyze the frequency of various combinations of the nucleobases A, C, U, and G in the sequence **S**_**t**_. In order to extract distinctive and discriminative features, the ${\varvec{XC}}$ matrices are subjected to normalization, resulting in the transformed matrices $\user2{ XC}^{\user2{^{\prime}}}$.

3 $${\varvec{XC}}^{\user2{^{\prime}}} = \frac{{\varvec{X}}}{{\mathop \sum \nolimits_{l = 0}^{q} \mathop \sum \nolimits_{m = 0}^{q} {\varvec{X}}\left( {l,m} \right)}}$$

Subsequently, the normalized co-occurrence matrix ${\varvec{XC}}^{\user2{^{\prime}}}$ is utilized to compute several properties, which include Entropy, Contrast, Energy, Homogeneity, and Dissimilarity^95,99–101^. The mathematical equations for these properties can be found in Table 3. These properties serve as quantitative measures to characterize different aspects of the co-occurrence patterns captured in the matrix ${\varvec{XC}}^{\user2{^{\prime}}}$. Afterwards, the features outlined in Table 3 are computed for each co-occurrence matrix Tables (ST1–ST8), and the corresponding feature vectors are presented in Table 4. Consequently, these feature vectors are utilized to construct the final feature set representation, denoted as **f**_**Set**_, for an RNA sequence of a miRNA sequence **S**_**t**_:

$${\mathbf{f}}_{{{\mathbf{Set}}}} \, = \,\left( {{\mathbf{f}}_{{\text{I}}} ,{\mathbf{f}}_{{\text{J}}} ,{\mathbf{f}}_{{\text{K}}} ,{\mathbf{f}}_{{\text{L}}} ,{\mathbf{f}}_{{\text{M}}} ,{\mathbf{f}}_{{\text{N}}} ,{\mathbf{f}}_{{\text{O}}} ,{\mathbf{f}}_{{\text{P}}} } \right).$$

**Table 3:** Features extracted from a co-occurrence matrix ${\varvec{XC}}^{\user2{^{\prime}}}$ of miRNA sequence **S**_**t**_. ${\varvec{XC}}^{\user2{^{\prime}}}$.

Feature	Mathematical formula
Energy	$\mathop \sum \limits_{l = 0}^{q} \mathop \sum \limits_{m = 0}^{q} {\varvec{XC}}^{\user2{^{\prime}}} \left( {l,m} \right)^{2}$
Entropy	$\mathop \sum \limits_{l = 0}^{q} \mathop \sum \limits_{m = 0}^{q} - {\varvec{XC}}^{\user2{^{\prime}}} \left( {l,m} \right){\text{ X }}\ln ({\varvec{XC}}^{\user2{^{\prime}}} \left( {l,m} \right))$
Homogeneity	$\mathop \sum \limits_{l = 0}^{q} \mathop \sum \limits_{m = 0}^{q} \frac{{{\varvec{XC}}^{\user2{^{\prime}}} \left( {l,m} \right)}}{{\left( {1 + \left( {l - m} \right)^{2} } \right)}}$
Contrast	$\mathop \sum \limits_{l = 0}^{q} \mathop \sum \limits_{m = 0}^{q} {\varvec{XC}}^{\user2{^{\prime}}} \left( {l,m} \right){\text{ X }}\left( {l - m} \right)^{2}$
Dissimilarity	$\mathop \sum \limits_{l = 0}^{q} \mathop \sum \limits_{m = 0}^{q} {\varvec{XC}}^{\user2{^{\prime}}} \left( {l,m} \right){\text{ X }}\left| {\left( {l - m} \right)} \right|$

**Table 4:** Extracted Feature vectors from the cooccurrence matrices.

Feature vector	Co-occurrence matrix
**f**_I_ = (f1, f2, f3, f4, f5)	**I** (Table ST1)
**f**_J_ = (f6, f7, f8, f9, f10)	**J** (Table ST2)
**f**_K_ = (f11, f12, f13, f14, f15)	**K** (Table ST3)
**f**_L_ = (f16, f17, f18, f19, f20)	**L** (Table ST4)
**f**_M_ = (f21, f22, f23, f24, f25)	**M** (Table ST5)
**f**_N_ = (f26, f27, f28, f29, f30)	**N** (Table ST6)
**f**_O_ = (f31, f32, f33, f34, f35)	**O** (Table ST7)
**f**_P_ = (f36, f37, f38, f39, f40)	**P** (Table ST8)

#### Shannon entropy

Shannon Entropy (*SE*) is a valuable metric for quantifying the information content or uncertainty within a given sequence. It assesses the entropy of information in a Bernoulli process where two possibilities (0/1) occur with a probability of $p$ ^102–105^. The *SE* signifies the degree of uncertainty present in a binary string and can be computed using the following formula:

4 $$SE = - \mathop \sum \limits_{i = 0}^{1} p_{i} \log_{2} (p_{i} )$$

where $p_{i}$ represents the probability of a binary sequence having two distinct values. When $p$ = 0, indicating that the event is impossible, there is no ambiguity, and the *SE* is 0. Likewise, when $p$ = 1, indicating a certain outcome, the *SE* is also 0. In the case where $p$ = 1/2^106^, the level of uncertainty is at its highest, resulting in an *SE* value of 1.

#### Hurst exponent

Hurst Exponent (*HE*) is a measure that characterizes the autocorrelation properties of a time series^107^ and finds applications in applied mathematics. It takes values between 0 and 1, where values in the range of [0, 0.5] indicate negative autocorrelation in the time series^108–110^. Positive autocorrelation, on the other hand, is indicated by values in the range of [0.5, 1]. A *HE* value of 0.5 suggests that the variable is uncorrelated with its previous values, indicating a random series. *HE* score increases with the strength of the correlation between successive values. The following equation is used to calculate the *HE* of a binary sequence *D* of length $n$, where $D_{i}$ represents the *i*^th^ element of the binary sequence *D*.

5 $$\frac{\Phi \left( n \right)}{{{\text{V}}\left( n \right)}} = \left( \frac{n}{2} \right)^{HE}$$

where

6 $$\Phi \left( n \right) = \max \left( {Y_{1} \ldots Y_{n} } \right) - \min \left( {Y_{1} \ldots Y_{n} } \right)$$

7 $$V\left( n \right) = \sqrt {\frac{1}{n}\left[ {\mathop \sum \limits_{i = 1}^{n} \left( {D_{i} - \upmu } \right)^{2} } \right]}$$

8 $$Y_{t} = \mathop \sum \limits_{i = 1}^{t} \left( {D_{i} - \upmu } \right), \forall t = 1, 2, 3 \ldots n$$

9 $$\upmu = \frac{1}{n} \mathop \sum \limits_{i = 1}^{n} D_{i}$$

#### Fractal dimension

The Fractal Dimension (*FD*) of miRNA sequences is a widely used feature for analyzing their structural complexity. The first step in calculating the *FD* involves transforming each miRNA sequence into indicator matrices^111,112^. The four nucleotides {A, U, C, G}c are represented by the symbol ${\tilde{\text{T}}}_{{{\text{miRNA}}}}$, and $D_{N}$ represents a miRNA sequence of length $N$ composed of four symbols chosen from ${\tilde{\text{T}}}_{{{\text{miRNA}}}}$. The indicator function for each miRNA sequence is defined by the following equation:

10 $$F:{ }D_{N} \times D_{N} \to \left\{ {0,1} \right\}, and{ }D_{N} = \left\{ {0,1} \right\}$$

Here the indicator matrix will be:

11 $$I\left( {N,N} \right) = \left\{ {\begin{array}{*{20}c} {1, s_{i} = s_{j} } \\ {0, s_{i} \ne s_{J} } \\ \end{array} } \right. \quad where\;s_{i} , s_{j} \epsilon D_{N}$$

To convert the miRNA sequence into a binary representation, a 2D dot-plot image is generated using the $I\left( {N,N} \right)$ matrix, which consists of values 0 and 1. This binary image visually represents the distribution of zeros and ones in the sequence, where white dots represent 0 and black dots represent 1. The *FD* can be computed from an indicator matrix by averaging the sigma $\sigma \left( k \right)$ values of 1 randomly selected from an $N$ × $N$ indicator matrix^112–114^. The following equation is used to calculate the *FD* based on the sigma $\sigma \left( k \right)$ value:

12 $$FD = - \frac{1}{N}\mathop \sum \limits_{k = 2}^{N} \frac{\log (\sigma \left( k \right))}{{\log k}}$$

### Machine learning and deep learning classifiers

In this comprehensive data analysis, we developed a total of *fourteen* ML models, including *nine* SML models and *five* EML models. Additionally, we constructed 24 DL models, consisting of *six* SDL models, *twelve* HDL models, and six EDL models.

#### Machine learning classifiers

For simplicity and availability, we selected the following ML models: LR^115,116^, Linear SVM^117–119^, Decision Tree (DT)^120^, RF^121–123^, Extra Trees (ET)^124,125^, Extreme Gradient Boost (XGBoost)^88,126^, K-Nearest Neighbors (KNN)^127,128^, Linear Discriminant Analysis (LDA)^129,130^, Light Gradient Boosting Machine (LGBM)^131,132^, and Naive Bayes (NB)^133^. We specifically chose six nonlinear models (DT, RF, ET, XGBoost, KNN, LGBM) as they are suitable for nonlinear classification tasks, which is crucial for effectively classifying binary-encoded miRNA species. Each model possesses unique strengths and weaknesses, and by evaluating multiple models, we can compare their performances and select the most effective one. Furthermore, we created five EML models: (i) LR and SVM, (ii) DT and KNN, (iii) DT and RF, (iv) RF, DT, and ET, and (v) ET, XGBoost, and LGBM. These models were constructed using a voting-based ensemble classifier approach.

#### Solo deep learning classifiers

Among the DL models, we developed *six* SDL models: GRU (Gated Recurrent Unit), Bidirectional GRU (BiGRU), RNN (Recurrent neural network), Bidirectional RNN (BiRNN), LSTM, and Bidirectional LSTM (BiLSTM). These models were specifically designed to capture the temporal dependencies and intricate patterns present in the miRNA sequences, further enhancing the classification performance. We conducted rigorous evaluation and testing to assess the performance and effectiveness of each SDL model, for the selection of the most suitable architecture for miRNA species classification.

#### Hybrid deep learning classifiers

While these SDL models have shown limited success in miRNA classification, combining them into HDL models has proven to be beneficial in overcoming data scarcity and improving performance^82,84,134,135^. HDL models can effectively address domain-specific challenges and enhance accuracy in tasks such as miRNA classification by leveraging multiple architectural components. Considering these advantages, we constructed twelve HDL models: (i) LSTM-GRU, (ii) BiLSTM-BiGRU, (iii) LSTM-CNN, (iv) BiLSTM-CNN, (v) GRU-CNN, (vi) BiGRU-CNN, (vii) BiRNN-CNN, (viii) BiGRU-GRU, (ix) BiLSTM-LSTM, (x) BiRNN-RNN, (xi) RNN-CNN, and (xii) LSTM-GRU-CNN.

#### Ensemble deep learning classifiers

Furthermore, we created six EDL models: (i) BiLSTM-BiGRU and LSTM-GRU, (ii) BiLSTM-BiGRU and BiRNN-RNN, (iii) BiGRU-GRre U and LSTM-CNN, (iv) BiRNN-CNN and GRU-CNN, (v) BiLSTM-LSTM and RNN-CNN, and (vi) BiLSTM-CNN and BiGRU-CNN by concatenating their output vectors. By combining these multiple vectors, we can leverage the strengths and advantages of each individual model. The EDL models are depicted in Figures F1, F2, F3, F4, F5, and F6 in the supplementary material. All constituent models are utilized without their output layers and are truncated until the dropout layers. These model components are then concatenated using a concatenate layer and further employed as input to a dense layer network. Finally, the network is connected to a softmax layer for predicting the species.

### Hypertuning parameters and optimization

During the study, the models were trained using a batch size of 64. The loss function chosen for training was categorical cross-entropy, which is commonly used for multi-class classification tasks. This loss function quantifies the dissimilarity between the predicted and actual probability distributions^136,137^.

The objective is to minimize the discrepancy between these distributions, leading to a reliable system that generates predicted probabilities that closely align with the true distribution. Categorical cross-entropy ensures that the differences between all probabilities are minimized. The mathematical equation for categorical cross-entropy is provided below:

13 $${\text{L}}_{{{\text{CCE}}}} { = }\frac{{1}}{{\text{N}}}\mathop \sum \limits_{{\text{i = 1}}}^{{\text{N}}} \mathop \sum \limits_{{\text{c = 1}}}^{{{\text{TC}}}} {1}_{{{\text{y}}_{{\text{i}}} \epsilon {\text{TC}}_{{\text{c}}} { }}} {\text{loga}}_{{{\text{model}}}} {\text{(y}}_{{\text{i}}} \epsilon {\text{TC}}_{{\text{c }}} {)}$$

where N represents the total number of miRNA sequences, TC denotes the number of species categories, and $1_{{{\text{y}}_{{\text{i}}} \epsilon {\text{TC}}_{{\text{c}}} }}$ indicates that the ^h^ observation belongs to the c^th^ category. Table ST9 in the supplementary material provides details on the number of epochs, initial learning rates, and optimizers utilized for each EDL model. The implementation of the study was carried out using Python 3.8 and the TensorFlow framework. The system execution occurred on a machine that featured a 12 GB NVIDIA P100 16 Graphics Processing Unit (GPU), an Intel Xeon Processors processor, and 12 GB of RAM.

### Performance metrics

The proposed models were assessed for both binary and multiclass classification tasks, with the multiclass approach utilizing the "one *vs*. all" strategy^138,139^ for each species. To evaluate the models, several parameters were considered: true positive (TP), true negative (TN), false positive (FP), and false negative (FN). A sample belonging to a specific species is considered a TP if it is correctly classified as such. Likewise, a sample not belonging to the species is labeled as a TN if it is correctly classified as not belonging. However, if a sample not belonging to the species is incorrectly classified as belonging, sample not belonging to the species is incorrectly classified as belonging, it is a FP, and if a sample belonging to the species is incorrectly classified as not belonging, it is a FN. These parameters allow the derivation of various performance evaluation (PE) metrics, including: (i) *Accuracy* (η): Indicates the proportion of correct overall predictions out of the total predictions made. (ii) *Recall* (R): Represents the ratio of correctly predicted positive class instances to all positive members in the dataset. (iii) *Precision* (P): Measures the ratio of correctly predicted positive class instances to the total number of classified positive predictions. (iv) *F1-score (F)*: The F1-score is the harmonic mean of precision and recall, serving as a valuable metric for evaluating model performance, especially on imbalanced datasets. (v) *Area-under-the-curve* ($\upalpha$): It quantifies the two-dimensional area beneath the plotted ROC curve and is commonly used to assess model performance in both binary and multiclass classification problems.

In this study, we introduce formulations to measure the overall robustness of the model. To achieve this, six quantities are measured in this section, including $\overline{\upeta }\left( {\text{m, K10}} \right)$, which represents the accuracy of model m summarized over all D datasets, $\overline{\upeta }\left( {\text{d, K10}} \right)$, which indicates the accuracy of dataset d achieved by summarizing M models, $\overline{\upeta }_{{{\text{sys}}}}$, which represents the overall system accuracy achieved by averaging accuracy over M models and D datasets, $\overline{\upalpha }\left( {\text{m, K10}} \right)$, which summarizes the AUC of model m over all D datasets, $\overline{\upalpha }\left( {\text{d, K10}} \right)$, which indicates the robustness of dataset d achieved by summarizing the AUC over M models, and $\overline{\upalpha }_{{{\text{sys}}}}$, which represents the overall system robustness achieved by averaging the AUC over M models and D datasets. These formulations are measured in each section for a combination of ML, SDL, HDL, and EDL models, as well as a combination of six binary and four multiclass datasets and their combinations. All these formulas were computed using the default K10 partition protocol.

14 $$\upeta = \frac{{\text{TP + TN}}}{{\text{TP + FP + FN + TN}}}$$

15 $${\text{R}} = \frac{{{\text{TP}}}}{{\text{TP + FN}}}$$

16 $${\text{P}} = \frac{{{\text{TP}}}}{{\text{TP + FP}}}$$

17 $$F = 2* \frac{{\text{P * R}}}{{\text{P + R}}}$$

18 $$\overline{\upeta }\left( {\text{m, K10}} \right){ = }\frac{{\mathop \sum \nolimits_{{\text{d = 1 }}}^{{\text{D}}} \upeta \left( {\text{m, d, K10}} \right)}}{{\text{D}}}{ }$$

19 $$\overline{\upeta }\left( {\text{d, K10}} \right){ = }\frac{{\mathop \sum \nolimits_{{\text{m = 1}}}^{{\text{M}}} { }\upeta \left( {\text{m, d, K10}} \right)}}{{\text{M}}}$$

20 $$\overline{\upeta }_{{{\text{sys}}}} { = }\frac{{\mathop \sum \nolimits_{{\text{d = 1 }}}^{{\text{D}}} \mathop \sum \nolimits_{{\text{m = 1}}}^{{\text{M}}} { }\upeta \left( {\text{m, d, K10}} \right)}}{{{\text{M }} \times {\text{D}}}}$$

21 $$\overline{\upalpha }\left( {\text{m, K10}} \right){ = }\frac{{\mathop \sum \nolimits_{{\text{d = 1 }}}^{{\text{D}}} \upalpha \left( {\text{m, d, K10}} \right)}}{{\text{D}}}$$

22 $$\overline{\upalpha }\left( {\text{d, K10}} \right){ = }\frac{{\mathop \sum \nolimits_{{\text{m = 1}}}^{{\text{M}}} { }\upalpha \left( {\text{m, d, K10}} \right)}}{{\text{M}}}$$

23 $$\overline{\upalpha }_{{{\text{sys}}}} { = }\frac{{\mathop \sum \nolimits_{{\text{d = 1 }}}^{{\text{D}}} \mathop \sum \nolimits_{{\text{m = 1}}}^{{\text{M}}} { }\upalpha \left( {\text{m, d, K10}} \right)}}{{{\text{M}} \times {\text{D}}}}$$

### Experimental protocols

To verify our hypothesis, we trained *nine* SML, *six* EML, *six* SDL, *twelve* HDL, and *six* EDL models, totalling 38 AI models, using a composite feature set. The feature set consisted of conventional features, including Entropy, Dissimilarity, Energy, Homogeneity, and Contrast, as well as contemporary features, such as Shannon entropy, Hurst exponent, and Fractal dimension. To test the resilience of the features on the AI models, we created various subsets of data with ten different datasets (six binary class and four multiclass).

#### Experiment 1: EDL Models vs. HDL Models vs. SDL Models

The main objective of this study is to examine and compare the effectiveness of SDL, HDL, and EDL models in classifying species using miRNA sequences. To achieve this, we trained and evaluated the performance of 24 AI models: six SDL, twelve HDL, and six EDL. The models were trained and tested using six binary and four multiclass balanced composite feature datasets. To evaluate the performance of these 24 AI models, their predictions were averaged across all ten datasets (6 binary class and 4 multiclass), and a comprehensive comparison was performed. To ensure the reliability of the results, the experiment utilized the K10 Cross-Validation protocols.

#### Experiment 2: EDL Models with CNN layers vs. without CNN layers

This study focuses on examining and comparing the impact of employing CNN layers into EDL models for species classification using miRNA sequences. The training and evaluation were conducted on twelve AI models, comprising four CNN-Based HDL models and two Non-CNN-Based HDL models. The models were trained and tested using six binary and four multiclass balanced composite feature datasets. To evaluate the performance of these 6 AI models, their predictions were averaged across all ten datasets (6 binary class and 4 multiclass), and a comprehensive comparison was performed. To ensure the reliability of the results, the experiment utilized the K10 Cross-Validation protocols.

#### Experiment 3: EML Models vs. SML Models

The primary aim of this study is to assess and contrast the efficacy of EML models versus SML models in the classification of species using miRNA sequences. The training and evaluation process involved 14 AI models, including nine SML models and five EML models. The models were trained and tested using six binary and four multiclass balanced composite feature datasets. To evaluate the performance of these 14 AI models, their predictions were averaged across all ten datasets (6 binary class and 4 multiclass), and a comprehensive comparison was performed. To ensure the reliability of the results, the experiment utilized the K10 Cross-Validation protocols.

#### Experiment 4: EDL Models vs. EML Models

The final objective of this study is to evaluate and compare the advantages offered by EDL models over EML models in stratifying species using miRNA sequences. A total of *eleven* AI models were trained and evaluated, including *five* EML models and *six* EDL models. The models were trained and tested using six binary and four multiclass balanced composite feature datasets. To evaluate the performance of these AI models, their predictions were averaged across all *ten* datasets (6 binary class and 4 multiclass), and a comprehensive comparison was performed. To ensure the reliability of the results, the experiment utilized the K10 Cross-Validation protocols.

## Results

The protocols were employed to conduct tests on miRNA data from *ten* datasets, comprising of *six* binary class datasets and *four* multiclass datasets. The binary datasets included Human *vs*. Gorilla, Human *vs*. Rat, Human *vs*. Mouse, Mouse *vs*. Gorilla, Mouse *vs*. Rat, and Gorilla *vs*. Rat datasets. Additionally, there were four multiclass datasets, namely Human *vs*. All, Rat *vs*. All, Gorilla *vs*. All, and Mouse *vs*. All. To analyze the data, a total of *fourteen* ML models and *eighteen* DL models were utilized. The ML models consisted of *nine* SML models and *five* EML models. The DL models consisted of *six* SDL models, *twelve* HDL models and *six* EDL models The training process involved using the TensorFlow and Sklearn frameworks, and a Tesla P100 GPU on the K10 partition protocol was utilized for executing the training process. Experimental results were obtained based on these procedures.

### EDL models vs. HDL models vs. SDL models

In this experiment, we conducted a comparison of *six* SDL classifiers, *twelve* HDL models and *six* EDL models. The performance evaluation involved calculating the average mean accuracy (ACC) and area-under-the-curve (AUC) for all the models across ten datasets, consisting of six binary class datasets and four multiclass datasets. The binary datasets comprised Human *vs*. Gorilla, Human *vs*. Rat, Human *vs*. Mouse, Mouse *vs*. Gorilla, Mouse *vs*. Rat, and Gorilla *vs*. Rat, while the multiclass datasets included Human *vs*. All, Rat *vs*. All, Gorilla *vs*. All, and Mouse *vs*. All. The results of the experiment are presented in Tables ST10, ST11, ST12, and ST13 given in the supplementary material.

Table ST10 shows that the SDL4 classifier (BiLSTM) achieved the best performance among all SDL models, with an ACC/AUC of **90.06%/0.9112**. In Tables ST11 and ST12, the HDL2 classifier (BiLSTM-BiGRU) performed the best among all HDL models, with an ACC/AUC of **92.53%/0.9306**. Furthermore, in Table ST13, the EDL6 classifier (BiLSTM-CNN ⊕ BiGRU-CNN) achieved the highest performance among all HDL/EDL models, with an ACC/AUC of **93.38%/0.9407**. Table 5 presents the mean comparison, indicating that EDL/HDL classifiers outperformed SDL classifiers on all datasets. The mean accuracy and AUC differences between HDL and SDL across all datasets were **2.17%** and **2.4%**, respectively. The mean accuracy and AUC differences between EDL and HDL across all datasets were **2.01%** and **1.52%**, respectively. Additionally, the mean accuracy and AUC differences between EDL and SDL across all datasets were **4.18%** and **3.92%**, respectively.

**Table 5:** Comparison of SDL *vs.* HDL *vs.* EDL models.

Comparison for six binary classifiers and four multiclass classifiers of SDL, HDL and EDL Models												
Dataset	SDL		HDL		EDL		Absolute difference (%)					
	$\upeta_{{{\text{SDL}}}}$(%)	$\upalpha_{{{\text{SDL}}}}$[0–1]	$\upeta_{{{\text{HDL}}}}$(%)	$\upalpha_{{{\text{HDL}}}}$[0–1]	$\upeta_{{{\text{EDL}}}}$(%)	$\upalpha_{{{\text{EDL}}}}$[0–1]	$\upeta_{{{\text{D1}}}}$	$\upalpha_{{{\text{D1}}}}$	$\upeta_{{{\text{D2}}}}$	$\upalpha_{{{\text{D2}}}}$	$\upeta_{{{\text{D3}}}}$	$\upalpha_{{{\text{D3}}}}$
Binary Class (BC) Classification												
Human *vs.* Gorilla	89.31	0.8998	92.19	**0.9336**	95.29	0.9575	2.88	3.38	3.1	2.39	5.98	5.77
Human *vs.* Mouse	81.21	0.8203	82.99	0.8384	85.77	0.8636	1.78	1.81	2.78	2.52	4.56	4.33
Human *vs.* Rat	83.19	0.8397	85.57	0.8766	87.92	0.8851	2.38	3.69	2.35	0.85	4.73	4.54
Mouse *vs.* Gorilla	94.11	0.9518	96.68	0.9704	97.28	0.9737	2.57	1.86	0.6	0.33	3.17	2.19
Mouse *vs.* Rat	91.23	0.9212	94.3	0.9575	95.66	0.9735	3.07	3.63	1.36	1.6	4.43	5.23
Rat *vs.* Gorilla	92.31	0.9295	95.38	0.9611	96.03	0.9696	3.07	3.16	0.65	0.85	3.72	4.01
Mean of 6 BC	88.56	0.8937	91.19	0.923	92.99	0.9372	2.63	2.93	1.8	1.42	4.43	4.35
Multiclass (MC) Classification												
Human *vs.* All	85.1	0.8545	85.88	0.8738	88.65	0.8934	0.78	1.93	2.77	1.96	3.55	3.89
Gorilla *vs.* All	93.53	0.9421	95.95	0.9702	97.45	0.9765	2.42	2.81	1.5	0.63	3.92	3.44
Rat *vs.* All	89.43	0.9104	90.54	0.9174	93.06	0.939	1.11	0.7	2.52	2.16	3.63	2.86
Mouse *vs.* All	87.68	0.8947	89.3	0.9046	91.74	0.924	1.62	0.99	2.44	1.94	4.06	2.93
Mean of 4 MCC	88.94	0.9004	90.42	0.9165	92.73	0.9332	1.48	1.61	2.31	1.67	3.79	3.28
Binary class + Multiclass Classification												
Mean of 10 Classifiers	88.71	0.8964	90.88	0.9204	92.89	0.9356	**2.17**	**2.4**	**2.01**	**1.52**	**4.18**	**3.92**

$\upeta$ (%) represents accuracy and $\upalpha$ (0-1) represents AUC. $\upeta_{{{\text{SDL}}}}$: Mean accuracy of SDL models; $\upalpha_{{{\text{SDL}}}}$: Mean AUC of SDL models; $\upeta_{{{\text{HDL}}}}$: Mean accuracy of HDL models; $\upalpha_{{{\text{HDL}}}}$: Mean AUC of HDL models; $\upeta_{{{\text{EDL}}}}$: Mean accuracy of EDL models; $\upalpha_{{{\text{EDL}}}}$: Mean AUC of EDL models;$\upeta_{{{\text{D1}}}} \user2{ }$: Mean absolute accuracy difference (HDL *vs.* SDL); $\upeta_{{{\text{D1}}}} \left( {\text{\% }} \right){ = }\user2{ }\left| {\upeta_{{{\text{HDL}}}} - \upeta_{{{\text{SDL}}}} } \right|$; $\upalpha_{{{\text{D1}}}}$ : Mean absolute AUC difference (HDL *vs.* SDL); $\upalpha_{{\text{D1 }}} {\text{(\% ) = }}\left| {\upalpha_{{{\text{HDL}}}} - \upalpha_{{{\text{SDL}}}} } \right| \times {100}$; $\upeta_{{{\text{D2}}}}$: Mean absolute accuracy difference (EDL *vs.* HDL); $\upeta_{{{\text{D2}}}} {\text{(\% )}} = \user2{ }\left| {\upeta_{{{\text{EDL}}}} - \upeta_{{{\text{HDL}}}} } \right|$ $\upalpha_{{\text{D2 }}}$: Mean absolute AUC difference (EDL *vs.* HDL); $\upalpha_{{\text{D2 }}} {\text{(\% ) = }}\left| {\upalpha_{{{\text{EDL}}}} - \upalpha_{{{\text{HDL}}}} } \right|{ } \times 1{00}$; $\upeta_{{{\text{D3}}}}$: Mean absolute accuracy difference (EDL *vs.* SDL); $\upeta_{{{\text{D3}}}} {\text{(\% )}} = \left| {\user2{ }\upeta_{{{\text{EDL}}}} - \upeta_{{{\text{SDL}}}} } \right|$; $\upalpha_{{\text{D3 }}}$: Mean absolute AUC difference (EDL *vs.* SDL); $\upalpha_{{\text{D3 }}} {\text{(\% ) = }}\left| {\upalpha_{{{\text{EDL}}}} - \upalpha_{{{\text{SDL}}}} } \right|{ } \times {100}$.

Significant values are in [bold].

These results validate our hypothesis that HDL classifiers perform better due to the complex nature of miRNA. HDL models can capture intricate nonlinear relationships between input features and output labels by recursively splitting the data into smaller subsets, enabling accurate predictions. Furthermore, combining multiple models in EDL/HDL classifiers allows them to leverage the strengths of different models, leading to improved performance. The ability to customize and adjust these models based on specific problem domains further enhances their effectiveness.

### EDL models with CNN layers vs. EDL models without CNN layers

In this experiment, we conducted a comparison to assess the impact of adding CNN layers in the architecture of EDL models. Specifically, we evaluated the performance of *four* CNN-based EDL classifiers (EDL3, EDL4, EDL5, and EDL6) and *two* non-CNN-based EDL classifiers (EDL1 and EDL2) on *ten* datasets, comprising of six binary class datasets and four multiclass datasets. The evaluation metrics of average mean accuracy and AUC were calculated and reported in Table 6. The results of our experiment demonstrated that incorporating CNN layers in the EDL models significantly enhanced their classification performance. By utilizing feature extraction techniques, the models exhibited improved accuracy and AUC scores. The mean absolute difference in accuracy and AUC across all datasets, resulting from the feature extraction process using contemporary features, was found to be **0.73%** and **0.92%**, respectively. These findings validated our hypothesis that incorporating CNN layers in DL models can enhance their effectiveness in classifying miRNA sequences. This improvement stems from the ability of CNN layers to capture both temporal and spatial dependencies within the data, enabling the models to learn hierarchical representations. The combination of temporal and spatial information allows for more comprehensive and accurate classification of miRNA sequences.

**Table 6:** Comparison of EDL models with CNN *vs.* without CNN layers.

Comparison for six binary classifiers and four multiclass classifiers of DL Models with and without CNN Layers								
Dataset	woCNN		wCNN		Absolute difference (%)		wCNN > woCNN	
	$\upeta_{{{\text{woCNN}}}}$(%)	$\upalpha_{{{\text{woCNN}}}}$[0–1]	$\upeta_{{{\text{wCNN}}}}$(%)	$\upalpha_{{{\text{wCNN}}}}$[0–1]	$\upeta_{{{\text{C1}}}}$	$\upalpha_{{{\text{C1}}}}$	Acccuracy (%)	AUC (%)
Binary Class (BC) Classification								
Human *vs.* Gorilla	95.21	0.9528	95.33	0.9598	0.12	0.7	0.12% Increase	0.7% Increase
Human *vs.* Mouse	83.89	0.8435	86.7	0.8737	2.81	3.02	2.81% Increase	3.02% Increase
Human *vs.* Rat	86.89	0.8727	88.44	0.8914	1.55	1.87	1.55% Increase	1.87% Increase
Mouse *vs.* Gorilla	96.44	0.9712	97.7	0.9749	1.26	0.37	1.26% Increase	0.37% Increase
Mouse *vs.* Rat	95.58	0.9674	95.7	0.9765	0.12	0.91	0.12% Increase	0.91% Increase
Rat *vs.* Gorilla	95.85	0.9693	96.12	0.9697	0.27	0.04	0.27% Increase	0.04% Increase
Mean of 6 BC	92.31	0.9295	93.33	0.941	1.02	1.15	1.02% Increase	1.15% Increase
Multiclass (MC) Classification								
Human *vs.* All	88.44	0.8904	88.75	0.895	0.31	0.46	0.31% Increase	0.46% Increase
Gorilla *vs.* All	97.18	0.975	97.58	0.9772	0.4	0.22	0.4% Increase	0.22% Increase
Rat *vs.* All	92.9	0.932	93.14	0.9426	0.24	1.06	0.24% Increase	1.06% Increase
Mouse *vs.* All	91.61	0.9207	91.81	0.9256	0.2	0.49	0.2% Increase	0.49% Increase
Mean of 4 MCC	92.53	0.9295	92.82	0.9351	0.29	0.56	0.29% Increase	0.56% Increase
Binary class + Multiclass Classification								
Mean of 10 Classifiers	92.4	0.9295	93.13	0.9387	**0.73**	**0.92**	**0.73% Increase**	**0.92% Increase**

$\upeta$ (%) represents accuracy and $\upalpha$ (0-1) represents AUC. $\upeta_{{{\text{woCNN}}}}$: Mean accuracy of EDL models without CNN layers; $\upalpha_{{{\text{woCNN}}}}$: Mean AUC of EDL models without CNN layers; $\upeta_{{{\text{wCNN}}}}$: Mean accuracy of EDL models with CNN layers; $\upalpha_{{{\text{wCNN}}}}$: Mean AUC of EDL models with CNN layers; $\upeta_{{{\text{C1}}}} \user2{ }$: Mean absolute accuracy difference (with *vs.* without CNN layers); $\upeta_{{{\text{C1}}}} \left( {\text{\% }} \right){ = }\user2{ }\left| {\upeta_{{{\text{wCNN}}}} - \upeta_{{{\text{woCNN}}}} } \right|$; $\upalpha_{{{\text{C1}}}}$ : Mean absolute AUC difference (with *vs.* without CNN layers); $\upalpha_{{\text{C1 }}} {\text{(\% ) = }}\left| {\upalpha_{{{\text{wCNN}}}} - \upalpha_{{{\text{woCNN}}}} } \right| \times {100}$.

Significant values are in [bold].

### EML models vs. SML models

In this experiment, we conducted a comparison of *nine* SML classifiers and *five* EML models. The performance evaluation involved calculating the average mean accuracy and AUC for all the models across ten datasets, consisting of six binary class datasets and four multiclass datasets. The binary datasets comprised Human *vs*. Gorilla, Human *vs*. Rat, Human *vs*. Mouse, Mouse *vs*. Gorilla, Mouse *vs*. Rat, and Gorilla *vs*. Rat, while the multiclass datasets included Human *vs*. All, Rat *vs*. All, Gorilla *vs*. All, and Mouse *vs*. All. The results obtained from the experiment are presented in Tables ST14 and ST15 in the supplementary material. Table ST14 displays the performance results of the SML models, where the ET classifier achieved the highest performance with an ACC/AUC of **90.33%/0.9049**. It was followed by RF with an ACC/AUC of **89.31%/0.8922** and LGBM with an ACC/AUC of **88.06%/0.8896**. In Table ST15, the EML4 classifier (DT ⊕ RF ⊕ ET) demonstrated the best performance among all the EML models, achieving an ACC/AUC of **91.14%/0.9171**.

Table 7 presents the mean comparison, indicating that the EML classifiers outperformed the SML classifiers on all datasets. The average accuracy and AUC differences between EML and SML across all datasets were **6.24%** and **6.46%**, respectively. These findings validate our hypothesis that EML models perform better due to the complex nature of miRNA, as they can capture intricate nonlinear relationships by recursively partitioning the data into smaller subsets, enabling accurate predictions. The use of a voting classifier in EML models allows them to combine the strengths of different models, leading to improved performance.

**Table 7:** Comparison of SML *vs.* EML models.

Comparison for six binary classifiers and four multiclass classifiers of SML and EML Models						
Dataset	SML		EML		Absolute difference (%)	
	$\upeta_{{{\text{SML}}}}$(%)	$\upalpha_{{{\text{SML}}}}$[0–1]	$\upeta_{{{\text{EML}}}}$(%)	$\upalpha_{{{\text{EML}}}}$[0–1]	$\upeta_{{{\text{M1}}}}$	$\upalpha_{{{\text{M1}}}}$
Binary Class (BC) Classification						
Human *vs.* Gorilla	79.82	0.8044	88.32	0.8893	8.5	8.49
Human *vs.* Mouse	67.82	0.6975	74.69	0.7431	6.87	4.56
Human *vs.* Rat	76.13	0.7792	83.69	0.846	7.56	6.68
Mouse *vs.* Gorilla	84.23	0.8603	90.84	0.9179	6.61	5.76
Mouse *vs.* Rat	81.6	0.826	86.97	0.8792	5.37	5.32
Rat *vs.* Gorilla	85.18	0.8552	92.03	0.9273	6.85	7.21
Mean of 6 BC	79.13	0.8037	86.09	0.8672	6.96	6.35
Multiclass (MC) Classification						
Human *vs.* All	75.73	0.7514	79.12	0.8094	3.39	5.8
Gorilla *vs.* All	84.91	0.8464	91.39	0.9096	6.48	6.32
Rat *vs.* All	81.24	0.8113	86.94	0.8829	5.7	7.16
Mouse *vs.* All	78.95	0.7825	84.04	0.8546	5.09	7.21
Mean of 4 MCC	80.21	0.7979	85.37	0.8641	5.16	6.62
Binary class + Multiclass Classification						
Mean of 10 Classifiers	79.56	0.8014	85.8	0.866	**6.24**	**6.46**

$\upeta$ (%) represents accuracy and $\upalpha$ (0-1) represents AUC. $\upeta_{{{\text{SML}}}}$: Mean accuracy of SML models; $\upalpha_{{{\text{SML}}}}$: Mean AUC of SML models; $\upeta_{{{\text{EML}}}}$: Mean accuracy of EML models; $\upalpha_{{{\text{EML}}}}$: Mean AUC of EML models; $\upeta_{{{\text{M1}}}} \user2{ }$: Mean absolute accuracy difference (EML *vs.* SML); $\upeta_{{{\text{M1}}}} \left( {\text{\% }} \right){ = }\user2{ }\left| {\upeta_{{{\text{EML}}}} - \upeta_{{{\text{SML}}}} } \right|$; $\upalpha_{{{\text{M1}}}}$ : Mean absolute AUC difference (EML *vs.* SML); $\upalpha_{{\text{M1 }}} {\text{(\% ) = }}\left| {\upalpha_{{{\text{EML}}}} - \upalpha_{{{\text{SML}}}} } \right|{ } \times {100}$.

Significant values are in [bold].

### EDL models vs. EML models

In this experiment, we conducted a comparison of *five* EML classifiers and *six* EDL models. The performance evaluation involved calculating the average mean accuracy and AUC for all the models across ten datasets, consisting of *six* binary class datasets and *four* multiclass datasets. The binary datasets comprised Human *vs*. Gorilla, Human *vs*. Rat, Human *vs*. Mouse, Mouse *vs*. Gorilla, Mouse *vs*. Rat, and Gorilla *vs*. Rat, while the multiclass datasets included Human *vs*. All, Rat *vs*. All, Gorilla *vs*. All, and Mouse *vs*. All. Table 8 presents the mean comparison, indicating that the EDL classifiers outperformed the EML classifiers on all datasets. The average accuracy and AUC differences between EDL and EML across all datasets were **7.09%** and **6.96%**, respectively.

**Table 8:** Comparison of EML *vs.* EDL models.

Comparison for six binary classifier.s and four multiclass classifiers of EML and EDL Models								
Dataset	EML		EDL		Absolute difference (%)		EDL > EML	
	$\upeta_{{{\text{EML}}}}$(%)	$\upalpha_{{{\text{EML}}}}$[0–1]	$\upeta_{{{\text{EDL}}}}$(%)	$\upalpha_{{{\text{EDL}}}}$[0–1]	$\upeta_{{{\text{E1}}}}$	$\upalpha_{{{\text{E1}}}}$	Acccuracy (%)	AUC (%)
Binary Class (BC) Classification								
Human *vs.* Gorilla	88.32	0.8893	95.29	0.9575	6.97	6.82	6.97% Increase	6.82% Increase
Human *vs.* Mouse	74.69	0.7431	85.77	0.8636	11.08	12.05	11.08% Increase	12.05% Increase
Human *vs.* Rat	83.69	0.846	87.92	0.8851	4.23	3.91	4.23% Increase	3.91% Increase
Mouse *vs.* Gorilla	90.84	0.9179	97.28	0.9737	6.44	5.58	6.44% Increase	5.58% Increase
Mouse *vs.* Rat	86.97	0.8792	95.66	0.9735	8.69	9.43	8.69% Increase	9.43% Increase
Rat *vs.* Gorilla	92.03	0.9273	96.03	0.9696	4	4.23	4% Increase	4.23% Increase
Mean of 6 BC	86.09	0.8672	92.99	0.9372	6.9	7	6.9% Increase	7% Increase
Multiclass (MC) Classification								
Human *vs.* All	79.12	0.8094	88.65	0.8934	9.53	8.4	9.53% Increase	8.4% Increase
Gorilla *vs.* All	91.39	0.9096	97.45	0.9765	6.06	6.69	6.06% Increase	6.69% Increase
Rat *vs.* All	86.94	0.8829	93.06	0.939	6.12	5.61	6.12% Increase	5.61% Increase
Mouse *vs.* All	84.04	0.8546	91.74	0.924	7.7	6.94	7.7% Increase	6.94% Increase
Mean of 4 MCC	85.37	0.8641	92.73	0.9332	7.36	6.91	7.36% Increase	6.91% Increase
Binary class + Multiclass Classification								
Mean of 10 Classifiers	85.8	0.866	92.89	0.9356	**7.09**	**6.96**	**7.09% Increase**	**6.96% Increase**

$\upeta$ (%) represents accuracy and $\upalpha$ (0-1) represents AUC. $\upeta_{{{\text{EML}}}}$: Mean accuracy of EML models; $\upalpha_{{{\text{EML}}}}$: Mean AUC of EML models; $\upeta_{{{\text{EDL}}}}$: Mean accuracy of EDL models; $\upalpha_{{{\text{EDL}}}}$: Mean AUC of EDL models; $\upeta_{{{\text{E1}}}} \user2{ }$: Mean absolute accuracy difference (EML *vs.* EDL); $\upeta_{{{\text{E1}}}} \left( {\text{\% }} \right){ = }\user2{ }\left| {\upeta_{{{\text{EDL}}}} - \upeta_{{{\text{EML}}}} } \right|$; $\upalpha_{{{\text{E1}}}}$ : Mean absolute AUC difference (EML *vs.* EDL); $\upalpha_{{\text{E1 }}} {\text{(\% ) = }}\left| {\upalpha_{{{\text{EDL}}}} - \upalpha_{{{\text{EML}}}} } \right| \times {100}$.

Significant values are in [bold].

These findings validate our hypothesis that EDL models outperform EML models due to their ability to capture complex patterns and relationships in the data through multiple layers of non-linear transformations. This can be attributed to their complex architecture, which allows them to automatically learn hierarchical representations of miRNA data, capturing both local and global patterns.

## Performance evaluation

The evaluation process encompassed a comprehensive analysis of the models' performance, employing various visualization techniques such as ROC curves and bar charts to visualize the performance of the models. To ensure the system's stability, its robustness and model stability are evaluated through observing effect of training data size on classifiers. This allowed us to provides insight into the reliability and stability of the models and identify areas for improvement.

### Receiver operating curves, mean accuracy curves, and mean AUC for classifier models

**Figure 2:**
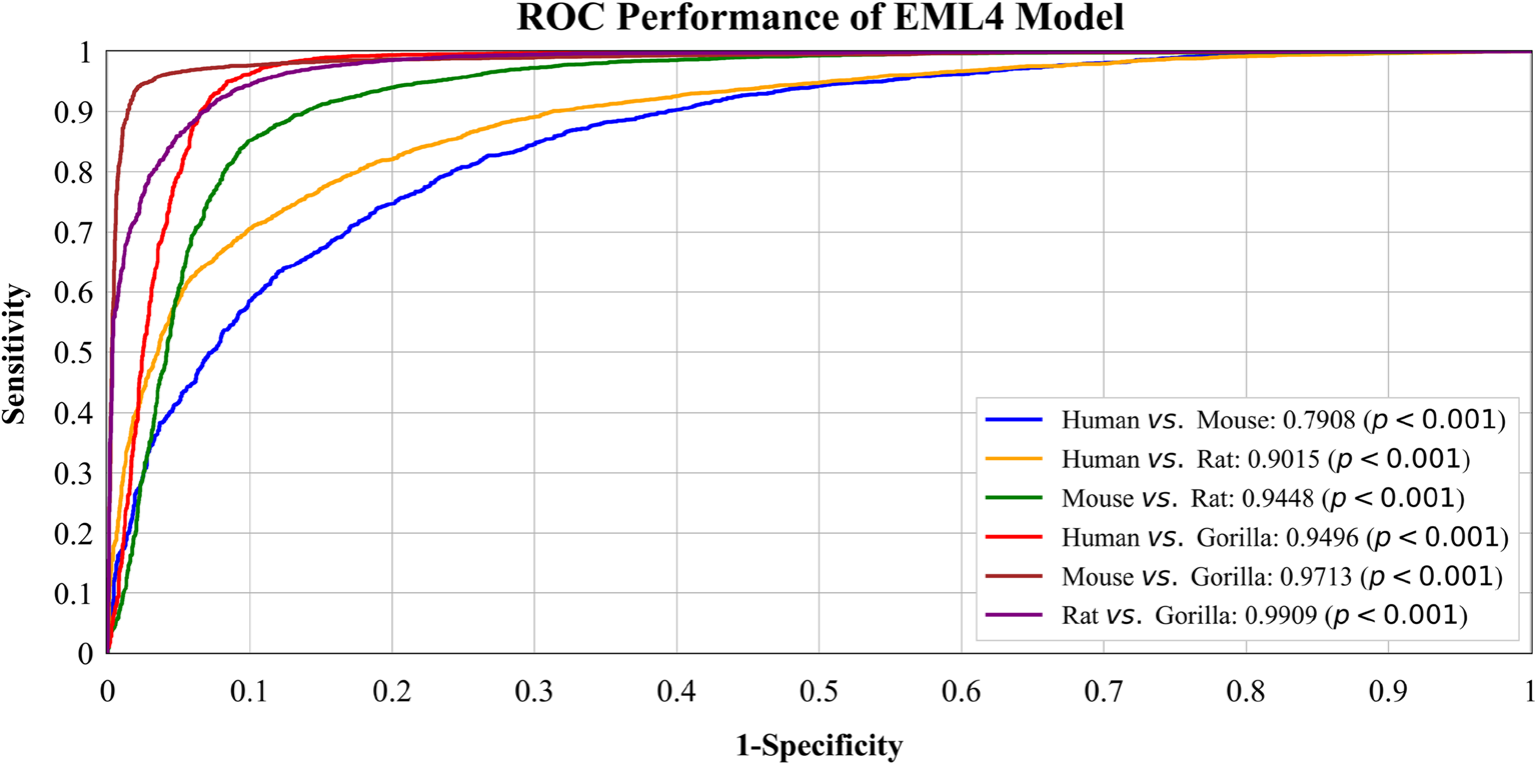
ROC curves for EML (EML4) model using K10 protocol.

**Figure 3:**
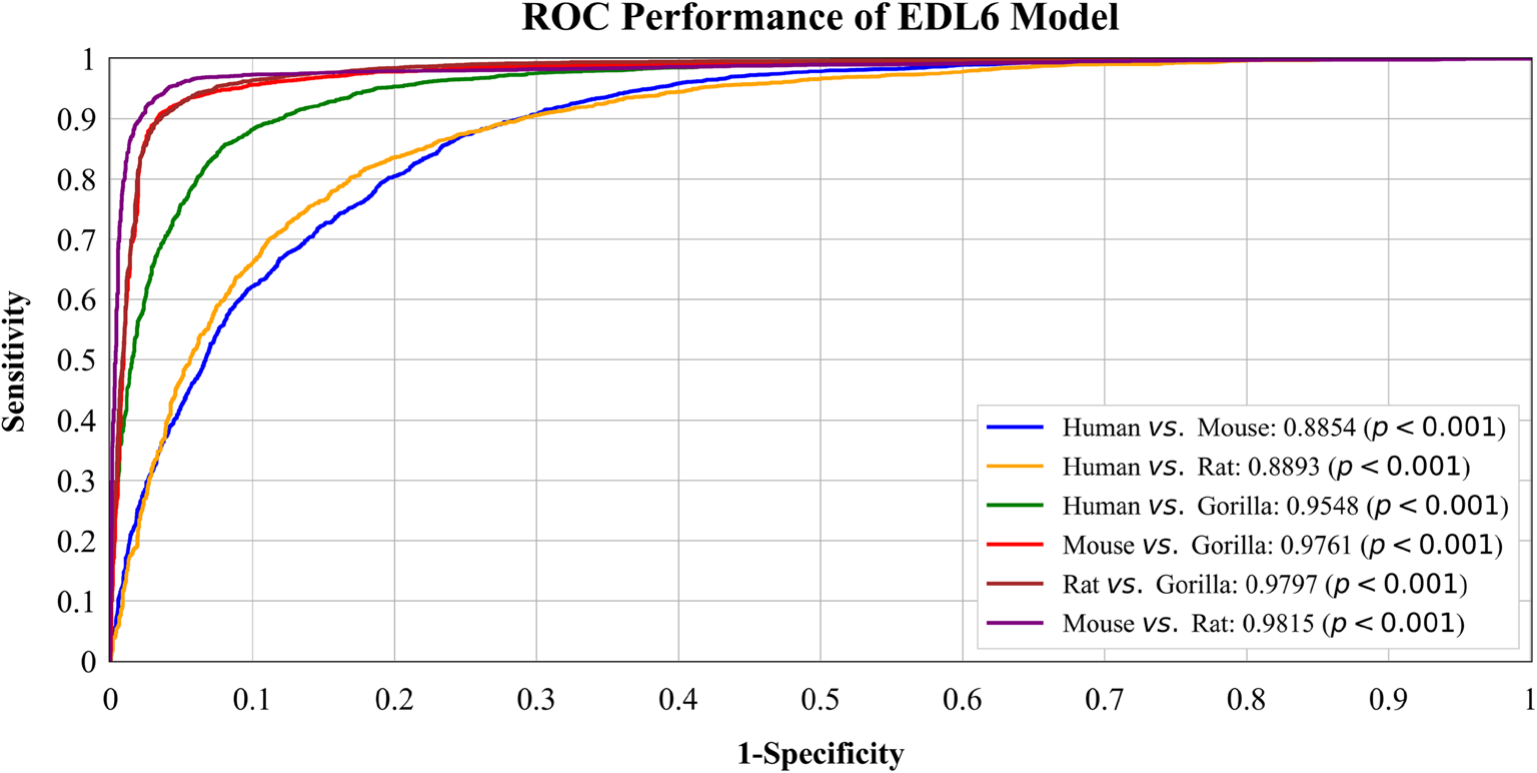
ROC curves for EDL (EDL6) model using K10 protocol.

We plotted ROC curves of two best models, with all their classifiers, on all *six* binary datasets: Human *vs*. Gorilla, Human *vs*. Rat, Human *vs*. Mouse, Mouse *vs*. Gorilla, Mouse *vs*. Rat, and Gorilla *vs*. Rat. The performance of the models across their complete operating range was thoroughly evaluated, as shown in Fig. 1. In Fig. 2, the ROC curve for the EML4 Model is presented. The AUC score for Rat *vs*. Gorilla is the highest at **0.9909**, followed by Mouse *vs*. Gorilla with an AUC score of **0.9713**. This is followed by Human *vs*. Gorilla with an AUC of **0.9496** and Mouse *vs*. Rat with an AUC of **0.9448**. The AUC score for Human *vs*. Rat is **0.9015**, and Human *vs*. Mouse has the lowest AUC score of **0.7908**. Figure 3 displays the ROC curve for the best-performing EDL (EDL6) Model. Among the comparisons, Mouse *vs*. Rat has the highest AUC score of **0.9815**, followed by Rat *vs*. Gorilla with an AUC score of **0.9797**. The AUC score for Mouse *vs*. Gorilla is **0.9761**, and Human *vs*. Gorilla has an AUC of **0.9548**. The AUC score for Human *vs*. Rat is **0.8893**, and Human *vs*. Mouse has the lowest AUC score of **0.8854**.

Furthermore, to establish the statistical significance of our results, *p*-values were computed for all species in each dataset. Our findings indicate that the *p*-values were less than **0.01**, signifying a high confidence level in the observed differences between the species.

**Figure 4:**
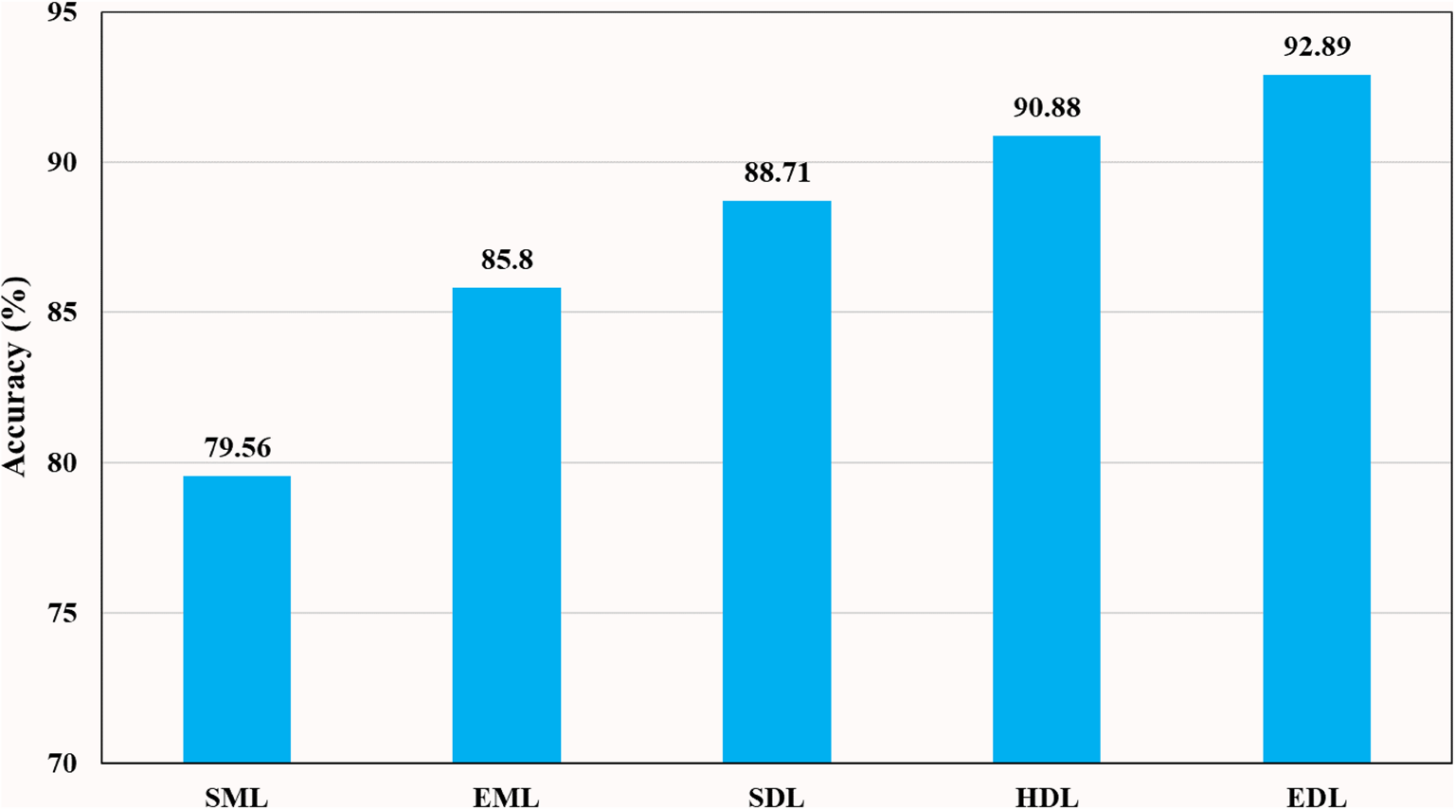
Comparison of Accuracy of SML *vs.* EML *vs.* SDL *vs.* HDL *vs*. EDL models using K10 protocol.

**Figure 5:**
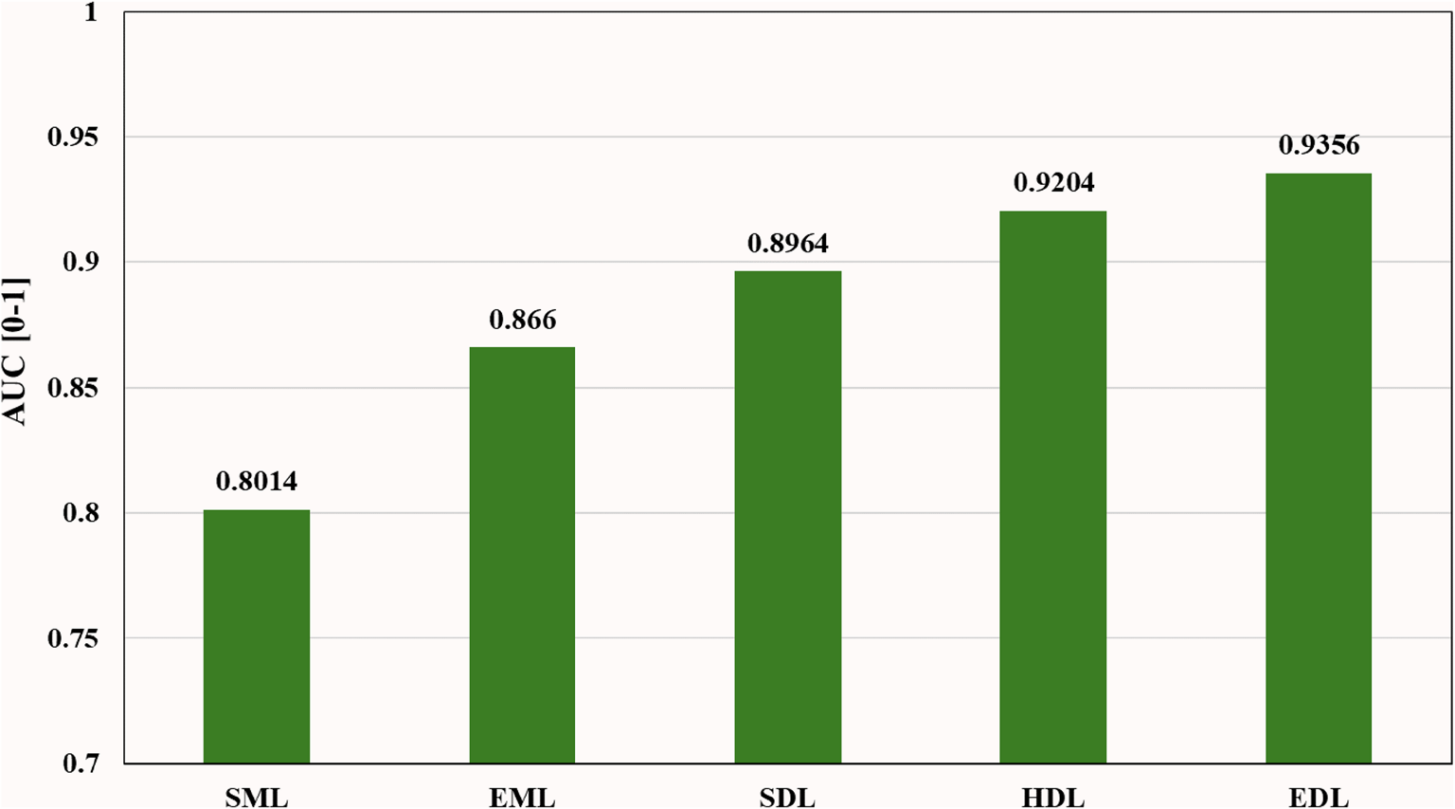
Comparison of AUC of SML *vs.* EML *vs.* SDL *vs.* HDL *vs*. EDL models using K10 protocol.

Bar charts are effective visual tools for presenting table data. Figure 4 illustrates the accuracy of *nine* SML, *five* EML, *six* SDL, *twelve* HDL, and *six* EDL models averaged across multiple binary and multiclass datasets. The mean accuracy increased progressively from **79.56%** (SML) to **85.8%** (EML), **88.71%** (SDL), **90.88%** (HDL), and **92.83%** (EDL) models. Additionally, Fig. 5 depicts the AUC of the same models, showing a similar progressive increase in mean accuracy from **0.8014** (SML) to **0.866** (EML), 0.**8964** (SDL), **0.9204** (HDL), and **0.933** (EDL) models when averaged across multiple binary and multiclass datasets.

### Effect of training data size on classifier performance: varying partitional protocols

In this experimental study, we investigated the influence of varying training data sizes on the performance of DL models. Performance metrics were evaluated using different Cross-Validation protocols, namely K10 (default), K5, K4, and K2. Our analysis, presented in Table 9, revealed a gradual decline in performance metrics across these protocols. The evaluation included 24 DL classifiers, consisting of 6 SDL, 12 HDL, and 6 EDL models, applied to ten datasets encompassing both binary class and multiclass datasets. The average mean accuracy and AUC were computed, indicating a decrease in mean accuracy from **90.82%** (K10) to **85.96%** (K2), corresponding to a **4.86%** reduction. Similarly, the AUC decreased from **0.9175** (K10) to **0.8634** (K2), indicating a **5.41%** decline. Despite the reduced amount of training data in the K2 (50:50) validation protocol, our DL models demonstrated reliable performance metrics. This finding emphasizes the effectiveness of our approach, particularly the benefits gained from using ensemble models along with feature extraction. Hence, our models exhibit strong performance even in scenarios with limited training data, demonstrating their ability to maintain consistent performance under such conditions.

**Table 9:** Mean performance of 24 DL models on different Cross-Validation protocols.

Performance Metrics	K2	Cross-Validation Results			Absolute Difference (%)		
		K4	K5	K10	D1	D2	D3
Accuracy (%)	85.96	87.2	88.54	90.82	**2.28**	**3.62**	**4.86**
AUC [0–1]	0.8634	0.8863	0.9019	0.9175	**1.56**	**3.12**	**5.41**
Recall (%)	86.06	87.73	88.98	91.39	2.41	3.66	5.33
Precision (%)	85.18	86.59	87.67	90.01	2.34	3.42	4.83
F1 Score (%)	85.62	87.16	88.32	90.69	2.37	3.53	5.07

**D1**: Absolute difference in performance metrics between K10 and K5 Cross-Validation protocols. **D1 = K10 -K5.**

**D2**: Absolute difference in performance metrics between K10 and K4 Cross-Validation protocols. **D2 = K10 -K4.**

**D3**: Absolute difference in performance metrics between K10 and K2 Cross-Validation protocols. **D3 = K10 -K2.**

Significant values are in [bold].

## Reliability analysis using statistical tests

The stability of the system was thoroughly assessed and validated using *three* statistical tests conducted on the EDL models across all *ten* testing sets. There are several published studies which uses statistical tests for establishing the reliability and stability of the AI system^80,81,140,141^. These tests are conducted on the employed models, and the specific tests we carried out are all showcased in the manuscript, namely Adjusted R2, Z (Two-Tailed), and ANOVA tests. The purpose of these tests was to determine the significance of the predicted data and monitor the *p*-value in the ANOVA test, ensuring it was less than 0.01 (*p* < 0.01). Detailed results of these tests, conducted following the methodology outlined in^96,142–145^, are presented in Table ST16 in the supplementary material. The outcomes revealed that all *six* EDL models (EDL1, EDL2, EDL3, EDL4, EDL5, and EDL6) exhibited statistical significance with *p* < 0.01 in the ANOVA test, indicating strong outcomes and highlighting the models' reliability, stability, and clinical importance. The adjusted R-squared test evaluated the accuracy of the models by measuring the extent of feature variance, while the Z-score in the two-tailed tests indicated the deviation of the score from the mean population in terms of standard deviation. Therefore, these statistically validated findings reinforce the significance of our results and provide strong support for the reliability of the EDL models in this study.

## Explainable artificial intelligence

To gain further insights into the decision-making process of the ML algorithms, we employed XAI techniques, specifically utilizing the SHapley Additive exPlanations (SHAP) method^146–150^. By leveraging SHAP, we were able to delve into the impact of different features on the classification outcomes, enhancing our understanding of species-specific information and the distinctive effects of individual features on each species. This invaluable information contributes to a deeper comprehension and differentiation among the various species.

**Figure 6:**
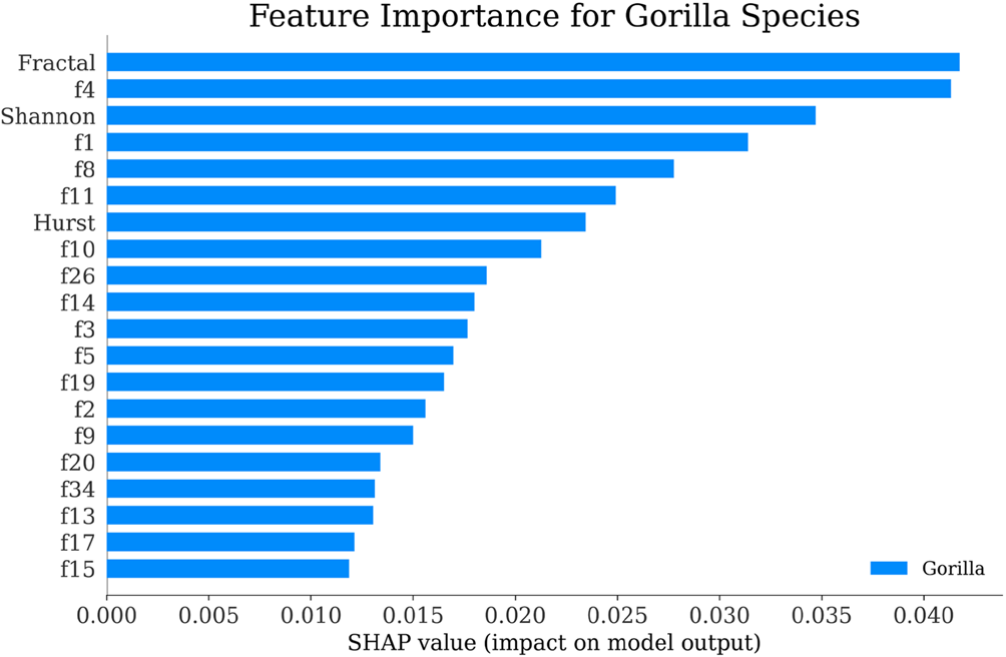
Feature Importance of Gorilla Species by SHAP explainer for EDL (EDL6) model using K10 protocol.

**Figure 7:**
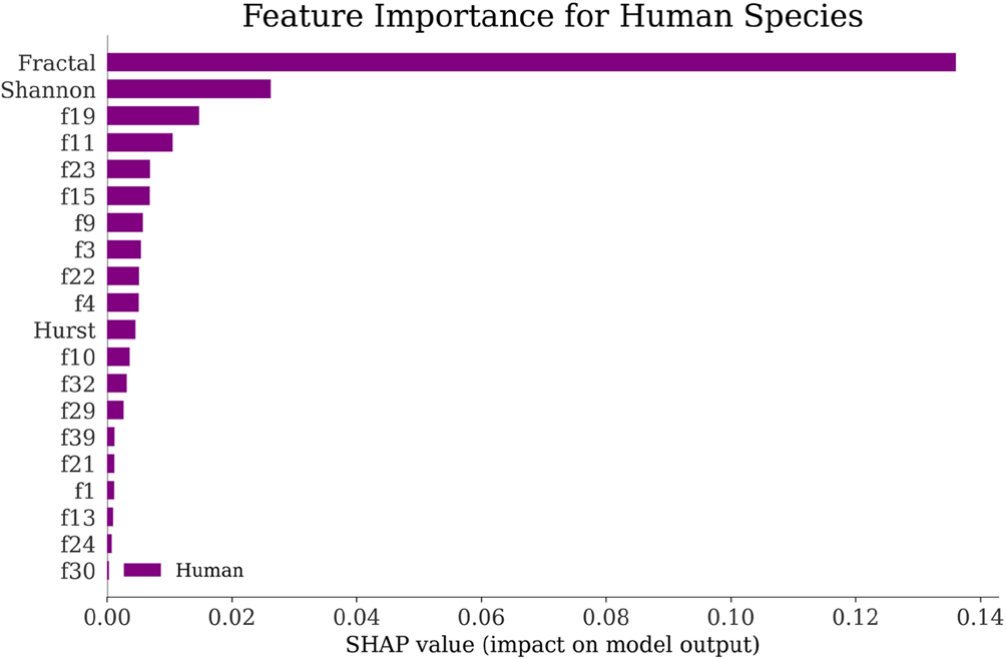
Feature Importance of Human Species by SHAP explainer for EDL (EDL6) model using K10 protocol.

**Figure 8:**
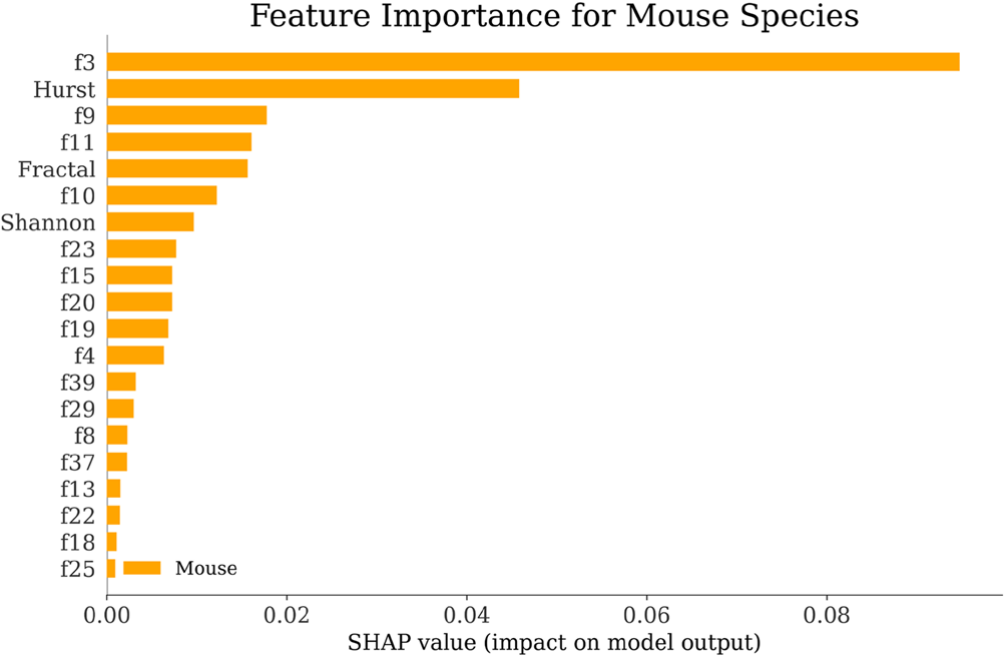
Feature Importance of Mouse Species by SHAP explainer for EDL (EDL6) model using K10 protocol.

**Figure 9:**
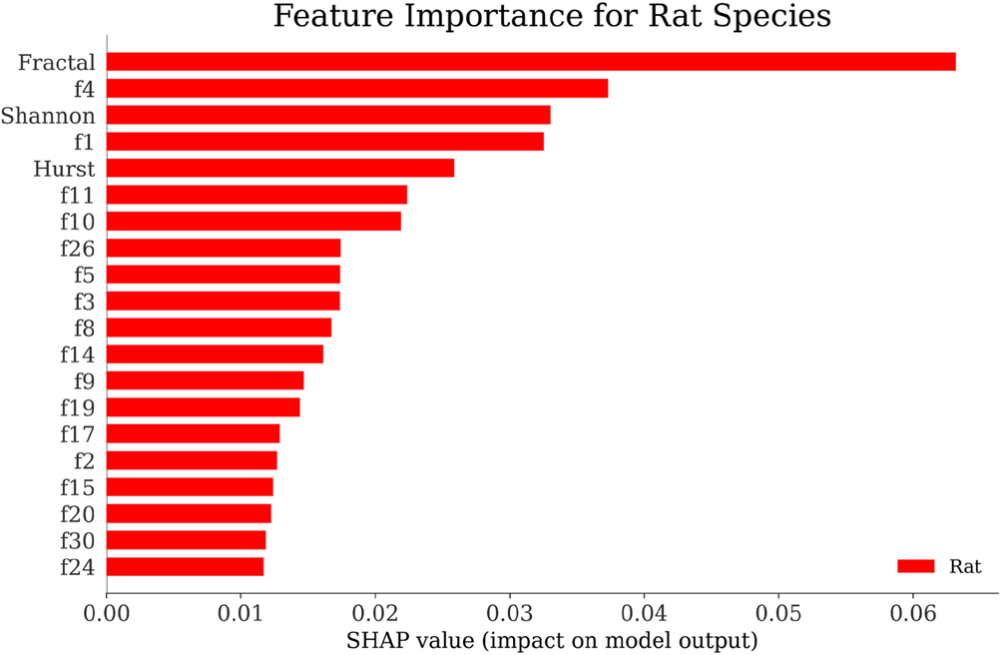
Feature Importance of Rat Species by SHAP explainer for EDL (EDL6) model using K10 protocol.

Using the SHAP explainer^151^, we developed an interpretable AI classifier as discussed in Fig. 1 that provided insights into the significance of different features for each species. The SHAP-generated graphs presented in Figs. 6, 7, 8 and 9 revealed that the "Fractal" feature played a crucial role in classifying all species except for the Mouse. In the case of the Mouse species, the most important feature was "f3," followed by "Hurst" and "f9." For the other three species, "Fractal" was the primary feature, accompanied by "Shannon" for Humans and "f4" for Gorilla and Rat. The importance of the remaining features, derived from the co-occurrence matrix as detailed in Table 4, gradually decreased. These findings emphasize the significance of feature selection when constructing accurate and dependable classifiers, particularly in biology and ecology^152^.

## Discussion

### Principal findings

After conducting an extensive study, we obtained valuable insights and drew conclusions pertaining to our research problem: (i) We devised four hypotheses and developed a total of 38 AI classifiers, which consisted of *nine* SML classifiers, *five* EML classifiers, *six* SDL classifiers, *twelve* HDL classifiers, and *six* EDL classifiers, in order to test them. (ii) For our experimental analysis, we utilized *ten* pre-processed datasets, comprising six binary classification datasets and four multiclass classification datasets. (iii) To enhance the processing and conversion of miRNA sequences into co-occurrence features, we implemented a novel quality control phase for our system. This involved performing scaling and binary encoding of the sequences. (iv) Our findings indicate that EML classifiers outperformed SML classifiers, yielding a mean accuracy increase of **6.24%** and a **6.46%** increase in AUC. Furthermore, HDL classifiers exhibited a significant advantage over SDL classifiers, with an increase in accuracy and AUC of **2.17%** and **2.4%**, respectively. (v) Also, EDL classifiers further improved upon HDL classifiers, with a mean accuracy of **2.01%** and an AUC of **1.52%**. (vi) Additionally, EDL classifiers significantly improved upon EML classifiers, with a mean accuracy increase of **7.09%** and an AUC of **6.96%**. (vii) We also observed that utilizing CNN-based HDL models with a feature extraction methodology greatly improved performance compared to non-CNN-based HDL models, yielding a mean accuracy increase of **0.73%** and a **0.92%** increase in AUC. (viii) We ensured the reliability and stability of our system by subjecting the classifiers to statistical tests. (ix) In order to verify the system's stability with smaller gene data sizes, we conducted a power analysis on the six binary class and four multiclass datasets, thereby validating the precision of the GeneAI 3.0 system. (x) We evaluated the impact of training data size by implementing Cross-Validation protocols in an increasing order. (xi) Finally, we utilized the SHAP explainer to interpret the classification results of the best (EDL6) model. This allowed us to gain insights into the significance of each species' features in their respective classifications.

### Benchmarking: a comparative analysis

Numerous methods have been suggested for miRNA classifiers and species-independent lncRNA predictors, such as Precursor miRNAs classification, Non-coding RNA classification, and cross-species miRNA identification. These methods have undergone extensive validation and proven effective in identifying and categorizing miRNA and lncRNA. In contrast, this study introduces a unique approach to classify miRNA based on stationary patterns derived from gene sequences. The primary aim is to determine the species of origin by analyzing specific parameters associated with each species family. While this approach is innovative, its effectiveness and practicality need to be assessed through a comparative analysis with existing methods. Comparing different approaches is crucial for advancing the field of miRNA classification and enhancing our comprehension of miRNA biology. Therefore, it is essential to evaluate the proposed approach's accuracy, efficiency, and generalizability in comparison to established methods.

Table 10 focused on six studies that focused on developing classifiers for miRNA and lncRNA. Yousef et al*.*^153^ employed a RF classifier and created a specific feature set called k-mer, which consisted of k-mer Distance, k-mer location distance, and k-mer first-last distance. These features were added to the basic k-mer features to classify Precursor miRNA. The evaluation of their method was conducted using a database obtained from USEARCH. Cao et al.^154^ explored the utilization of an RF model with incremental feature selection and the Pearson correlation coefficient. Their objective was to predict lncRNA from both lncRNA and mRNA transcripts in a dataset consisting of six species. The dataset used in their study was sourced from Ensemble data repository.

Gu et al*.*^155^ introduced an Ensemble Learning approach for miRNA-related disease classification using a multi-classifier system based on associated probabilities. Their method aimed to discover new potential associations between miRNA and diseases. The results were validated using various versions of the HMDD database, making it a reliable approach that does not rely on known associations between miRNA and diseases. Zhao et al.^156^, introduces an improved paradigm for miRNA target prediction was presented. They utilized a DT-based meta-strategy and a multi-threshold sequential voting method for meta-prediction. This approach aimed to enhance the accuracy of existing miRNA target prediction schemes.

Jiang et al.^157^ implemented a neural network-based scheme for end-to-end classification of pre-miRNA. They utilized a database consisting of 98 features, including n-gram frequency, structural sequence, structural diversity, and energy. The approach incorporated primary and secondary structure information to identify pre-miRNA in seven different species. Amin et al*.*^158^ employed a comprehensive feature extraction approach for non-coding RNA classification. They constructed an extensive feature database and trained it using LR and RF models. The database consisted of peptide features, open reading frame (ORF) features, and whole sequence features, with classifiers individually applied to each feature class. A hierarchical majority voting mechanism was utilized to combine the features.

**Table 10:** Benchmarking table showing studies that were implemented for miRNA and lncRNA classification.

Author & Year	Objective	Method	Model	Feature Extraction	Dataset	Class Type	Performance	CVP*	Clinical Validation
Yousef et al.^153^	Precursor miRNAs classification	Addition of K-mer Distance, K-mer Location Distance, and K-mer First-Last Distance to the core K-mer Features for Classification	RF	K-mer Distance Features	USEARCH (16 Species)	BC & MC	ACC: 93% ACC: 86% (Laurasiatheria)	K100 Monte Carlo	×
Jiang et al.^157^	Precursor miRNA classification	Backpropagation Neural network model was used to identify microRNA precursors using 98-dimensional novel features	ANN	Conventional Features	Carleton (SMIRP)	BC	ACC: 93.42%	K5	×
Cao et al.^154^	Predicting lncRNAs	Predicting lncRNA from lncRNA and mRNA transcripts, a RF classifier with incremental feature selection and the Pearson correlation coefficient was used	RF	Incremental feature selection	Ensembl v97, GreeNC (6 Species)CD-hit	Class Specific	ACC: 91.09%	K10	Adjusted *p*-value; Z-value
Zhao et al.^156^	Predicting lncRNAs	Improvement paradigm for miRNA target prediction using DT-based meta-strategy and multi-threshold sequential-voting	DT-based voting sytem	Multi-threshold sequential-voting for meta-prediction	MiRTarBase	BC	ACC: 91.09%	×	×
Gu et al.^155^	Predicting lncRNAs	Ensemble Learning based approach using a multi-classifer based system to miRNA related to disease by discovering new potential associations	Multi-classifiers voting	Similarity and structural feature data	HMDD V2.0	BC	AUC: 0.9229	K5	×
Amin et al.^158^	Non-coding RNA classification	Development of a Feature database of Peptide, ORF, and Whole sequence and selection using separate classifiers with Hierarchical majority voting	LR RF	Extensive Feature selection based on database, species and ncRNA type	RNACentral (16 Species)	BC	ACC: 91.928%(All Features) F1-score: 94.885% (All Features)	Nested K10	Chi-squared (in model)
Singh et al. Proposed	miRNA classification	Using Shannon Entropy, Hurst Exponent and Fractal Dimension along with contemporary features like Entropy, and Diversity to predict Species from MicroRNA gene sequence	ML SDL HDL EDL	Stationary Patterns of Nucleotides	miRNA Database (4 Species)	BC & MC	Best ACC (EDL): 97.41% Best EDL AUC: 0.97 (EDL1 in supplementary material)	K2, K4, K5, and K10 (Default)	R2, Z-two tailed, ANOVA

*CVP Cross-Validation Protocol; *ACC* Accuracy (%); *BC* Binary Class; *MC* Multiclass Classification; *XAI* Explainable AI; *ML* Machine Learning; *SDL* Solo Deep Learning; *HDL* Hybrid Deep Learning; *EDL* Ensemble Deep Learning; *LR* Logistic Regression; *RF* Random Forest; *CNN* Convolutional Neural Networks; *ANN* Artificial Neural Networks; K#: Cross-Validation protocol having the ratio of training: testing data sets; K2: 50%:50%; K4: 75%:25%; K5:80%:20%; K10: 90%:10%.

In our proposed work (R7), we introduce a novel approach for miRNA classification based on species of origin. Multiple LSTM, GRU, CNN, and RNN-based SML, EML, SDL, HDL, and EDL models are employed. A feature extraction module is used to extract both conventional features like entropy and energy, as well as contemporary features such as Shannon entropy, Hurst exponent, and fractal dimension. This integration of different features helps build a more robust model. Our study focuses on achieving generalization, employing XAI as part of scientific validation, and conducting thorough testing to ensure the reliability and stability of the GeneAI 3.0 system.

### Special note on ensemble-based feature extraction in miRNA classification

Ensemble-based feature extraction techniques have emerged as a powerful approach in miRNA classification tasks. By combining multiple feature extraction methods, using concatenation and splitting, these ensembles can effectively capture diverse aspects of miRNA sequences, leading to improved classification performance. The ensemble architecture allows for the fusion of features extracted from different methods, such as structural and compositional information, enabling the neural network to leverage complementary information and capture complex patterns in miRNA data. This approach not only enhances the classification accuracy but also helps mitigate overfitting by providing a regularization effect. Additionally, by incorporating different ensemble architectures, including completely different paradigms, the ensemble-based feature extraction further enriches the classification process, allowing for a more comprehensive and robust miRNA classification.

The effectiveness of ensemble-based techniques in miRNA classification is not limited to DL but also observed in traditional ML approaches. Techniques like RF and stacked ML models employ ensembles of multiple ML models to enhance classification performance. The ensemble architectures, such as weighted averaging, hard voting, and soft voting, play a crucial role in combining the predictions or features extracted from different models, leveraging their complementary strengths, and achieving better classification outcomes in miRNA analysis. By harnessing the collective intelligence of multiple models, ensemble-based feature extraction offers a powerful framework to improve the accuracy, sensitivity, and specificity of miRNA classification models. These ensemble-based approaches pave the way for more reliable and robust miRNA classification, enabling researchers to gain deeper insights into the complex world of gene expression and regulation.

### Special note on generalization

For generalizations, the models have to undergo training and testing on multiple datasets. Our group has done several methods for generalization^78–81^. In^81^, we developed an ensemble-based transfer learning paradigm, successfully classifying skin lesion images from two different and diverse datasets. We trained on one set and classified lesions from the other set. Study^80^ focused on our work in depression detection, where we developed a generalized model for text classification with the primary goal of detecting depression. Study^79^ attempted to achieve generalization in Covid-19 patients' lung segmentation across five different combinations of data by employing unseen data tests and statistical analyses. Finally, Study^78^, focusing on COVID-19 lung computed tomography segmentation, achieved generalization by testing on two unseen datasets, pairing 72 Italian and 80 Croatian patients.

We achieved generalization in these systems by simplifying the model, enabling it to work across multiple domains effectively in various situations through the mixing of domains. In Study^80^, we trained a model to be robust enough for depression detection as well as sentiment analysis by facilitating inter-dataset (cross-domain) training and leveraging knowledge from a multi-domain dataset. Our model demonstrated the capability to detect depression even when trained on a sentiment dataset, while also analysing sentiment when trained on a depression dataset. Likewise in this study, we conducted both multi-class and binary class classification, comprising a total of 10 datasets, where the model demonstrated satisfactory performance. This generalization ensures the effectiveness of our models for use in real-life scenarios, as any gene sequence can be pre-processed, features extracted and utilized by our model.

### Strengths, weakness, and extensions

Our study presents a novel approach to gene dataset analysis using 38 AI classifiers, which consisted of *nine* SML classifiers, *five* EML classifiers, *six* SDL classifiers, *twelve* HDL classifiers, and *six* EDL classifiers. Through rigorous evaluation, we found that these models demonstrated exceptional performance in both binary and multiclass classification tasks. Furthermore, our study involved building an extensive composite feature set, generating new features such as Shannon entropy, Hurst exponent, and Fractal dimension, which were incorporated with existing co-occurrence features to enhance the AI system's performance. Additionally, our study addressed the challenge of interpretability by incorporating XAI techniques, allowing us to gain insights into the inner workings of the models. This enables us to leverage feature-specific knowledge and concentrate on further research for each species independently. It provides a critical overview of the important features that individually impact the likelihood of a miRNA sequence belonging to a specific species. This knowledge, derived from the feature plots, is crucial for the practical implementation of the machine learning model in our study. It will significantly influence how we hypertune our model and, on a biological level, understand which features (both conventional and contemporary) matter more for each species. Notably, our proposed methodology demonstrated robustness through its consistent performance in multiple statistical tests, including the Adjusted R2 Test, paired T-test, ANOVA, and null-hypothesis significance testing (*p*-value). Across all six binary and four multiclass datasets, our methodology consistently provided interpretable, reliable and accurate results, highlighting its potential to improve classification accuracy in gene species classification.

One limitation of our gene classification approach is the potential for model generalization and overfitting due to the limited size of the available training data, especially in binary class classification tasks. Although ensemble-based models have been employed to mitigate this issue, there is room for improvement by utilizing Generative Adversarial training-based mechanisms to synthesize additional data. Another weakness is the absence of attention mechanisms, which could hinder the model's ability to mitigate overfitting and enhance its overall robustness. To address these limitations, incorporating attention-based techniques can offer a more focused and streamlined classification of species, ultimately improving the accuracy and reliability of the gene classification scheme.

In the future, we can further enhance our gene classification scheme by addressing limitations and implementing potential improvements. One major limitation is the lack of diversity in the dataset, which can hinder the model's ability to generalize. To overcome this, we can incorporate a wider range of gene species and sequences into the dataset. This can be achieved by leveraging big data sources^159^ or exploring other public data repositories^160,161^. By expanding the dataset, we can train more complex models that exhibit improved accuracy and generalization performance. In dealing with gene sequence data, graph neural networks and attention-enabled mechanisms show promise^162–164^. These approaches can better capture the intricate relationships between gene sequences and the species of origin. By leveraging these techniques, we can enhance the accuracy and interpretability of our gene classification scheme. To address the scarcity of data available for training models, we can consider employing Generative Adversarial training-based schemes. These schemes can generate synthetic data, thereby augmenting the training set and helping to overcome the data dearth^165–167^. We also plan to enhance our model by employing a cross-domain-based framework. This involves training on one gene sequence dataset and testing on another from a different database. More gene data can be selected, evaluated to prove the deep learning methods. Another avenue to explore is the utilization of Autoencoders in gene classification. Autoencoders have the ability to reduce dimensionality and extract essential features from the data. By incorporating an Autoencoder-based paradigm, we can improve the efficiency and accuracy of gene classification tasks^168–170^. Additionally, applying pruning strategies for AI models^141^ and studying the comorbidity effect in genomics can contribute to enhancing the classification system. Pruning techniques optimize the model's architecture and computational efficiency, while investigating comorbidity sheds light on the interconnected nature of genetic factors and disease manifestation^171^. By implementing these potential improvements, we can develop more accurate and robust models with broader applicability in the fields of genetics and bioinformatics.

## Conclusion

This study presents a novel paradigm for feature extraction in miRNA classification using EDL and EML models. Specifically, we utilized 38 types of AI models (*nine* SML, *six* EML, *six* SDL and *twelve* HDL and *six* EDL) architectures, to extract features from co-occurrence-based binary-coded sequences. The extracted composite features combined contemporary and conventional features, resulting in a total of 43 generated features. We conducted a thorough data analysis using 10 classification algorithms, including binary and multiclass classifiers, and four experimental protocols to evaluate the effectiveness of our proposed scheme. Our results showed that our proposed scheme outperformed existing methods regarding accuracy, sensitivity, and specificity. Furthermore, we conducted Cross-Validation to ensure the robustness of our model, and our results demonstrated that our model was highly reliable even with limited training data. Finally, we conducted statistical tests to demonstrate the reliability and stability of our Artificial Intelligence system.

### Supplementary Information

Supplementary Information.

## Data Availability

The datasets generated during and analyzed during the current study are not publicly available due to their propriety nature but are available from the corresponding author on reasonable request.

## Abbreviations

AI: Artificial intelligence
ACC: Accuracy
ADASYN: Adaptive synthetic sampling approach for imbalanced leaning
ANOVA: Analysis of variance
AUC: Area-under-the-curve
BC: Binary classification
BiGRU: Bidirectional GRU
BiLSTM: Bidirectional LSTM
BiRNN: Bidirectional RNN
CNN: Convolutional neural network
DL: Deep learning
DT: Decision trees
ET: Extra trees
EDL: Ensemble deep learning
EML: Ensemble machine learning
FD: Fractal dimension
FN: False negative
FP: False positive
GPU: Graphics processing unit
GRU: Gated recurrent unit
HDL: Hybrid deep learning
HE: Hurst exponent
HINN: Hierarchical input neural networks
KNN: K-nearest neighbors
LDA: Linear discriminant analysis
LGBM: Light gradient boosting model
lncRNA: Long non-coding RNAs
LR: Logistic regression
LSTM: Long short-term memory
MCC: Multiclass classification
miRNA: MicroRNA
ML: Machine learning
mRNA: Messenger RNA
NB: Naïve Bayes
ORF: Open reading frame
RF: Random forest
ROC: Receiver operating curves
RNA: Ribonucleic acid
RNN: Recurrent neural network
SDL: Solo deep learning
SHAP: Shapley additive explanations
SE: Shannon entropy
SML: Solo machine learning
SVM: Support vector machine
TP: True positive
TN: True negative
XAI: Explainable AI
Xgboost: Extreme gradient boost

## Symbols

$$\upeta$$: Accuracy
R: Recall
P: Precision
*F*: F1-score
$$\mu$$: Arithmetic mean
A: Adenine
U: Uracil
C: Cytosine
G: Guanine
${\varvec{XC}}$: Co-occurrence matrix
${\varvec{XC}}^{\prime }$: Normalized co-occurrence matrix
**S**_**t**_: MiRNA sequence
$n$: Length of miRNA sequence
$p$: Probability of Bernoulli process
$D_{N}$: Binary miRNA sequence
**f**_**Set**_: Final feature set representation
$\Phi \left( n \right)$: Difference between maximum and minimum instances of the binary miRNA sequence
$V\left( n \right)$: Standard deviation of the Binary miRNA sequence
$${\tilde{\text{T}}}_{{{\text{miRNA}}}}$$: Nucleotides representation: {A, U, C, G}
${\text{L}}_{{{\text{CCE}}}}$: Categorical cross-entropy Loss
$\overline{\upeta }\left( {{\text{m}},{\text{K}}10} \right)$: Accuracy of model ‘m’ summarized over all D datasets over K10 protocol
$\overline{\upeta }\left( {\text{d, K10}} \right)$: Accuracy achieved over dataset ‘d’ over all M Models over K10 protocol
$\overline{\upeta }_{{{\text{sys}}}}$: Overall system accuracy over M models and D datasets
$\overline{\upalpha }\left( {\text{m, K10}} \right)$: AUC of model m summarized over all D datasets
$\overline{\upalpha }\left( {\text{d, K10}} \right)$: AUC achieved over dataset d over all M Models
$\overline{\upalpha }_{{{\text{sys}}}}$: Overall system AUC over M models and D datasets
M: Total number of Models used in study
D: Total number of Datasets used in study
⊕: Concatenation of two models

## Code availability

The code used during the current study are not publicly available due to due to their propriety nature but are available from the corresponding author on reasonable request.
