# Supplementary material for: GeneAI 3.0: powerful, novel, generalized hybrid and ensemble deep learning frameworks for miRNA species classification of stationary patterns from nucleotides
Source: Sci Rep. 2024 Mar 26;14:7154. doi: 10.1038/s41598-024-56786-9 (PMC11344070; doi:10.1038/s41598-024-56786-9)
Supplement: Supplementary file 1 — Supplementary Information. [file 41598_2024_56786_MOESM1_ESM.docx]

**GeneAI 3.0: Powerful, Novel, Generalized Hybrid and Ensemble Deep Learning Frameworks for miRNA Species Classification of Stationary Patterns from Nucleotides**

**Supplementary Materials**

**A. Tables**

Table ST1. Co-occurrence matrix **I** containing the patterns of nucleobases^*^ in the miRNA sequence.

|  | A | C | U | G |
| --- | --- | --- | --- | --- |
| A | #(AA) | #(AC) | #(AU) | #(AG) |
| C | #(CA) | #(CC) | #(CU) | #(CG) |
| U | #(UA) | #(UC) | #(UU) | #(UG) |
| G | #(GA) | #(GC) | #(GU) | #(GG) |

^*^Four strands; A: Adenine; C: Cytosine; U: Uracil; G: Guanine.

Table ST2. Co-occurrence matrix **J** containing the pattern of nucleobases^*^ in the miRNA sequence.

|  | A | C | U | G |
| --- | --- | --- | --- | --- |
| AA | #(AAA) | #(AAC) | #(AAU) | #(AAG) |
| CC | #(CCA) | #(CCC) | #(CCU) | #(CCG) |
| UU | #(UUA) | #(UUC) | #(UUU) | #(UUG) |
| GG | #(GGA) | #(GGC) | #(GGU) | #(GGG) |

^*^Four strands; A: Adenine; C: Cytosine; U: Uracil; G: Guanine.

Table ST3. Co-occurrence matrix **K** containing the pattern of nucleobases^*^ in the miRNA sequence.

|  | A | C | U | G |
| --- | --- | --- | --- | --- |
| AC | #(ACA) | #(ACC) | #(ACU) | #(ACG) |
| AU | #(AUA) | #(AUC) | #(AUU) | #(AUG) |
| AG | #(AGA) | #(AGC) | #(AGU) | #(AGG) |
| CU | #(CUA) | #(CUC) | #(CUU) | #(CUG) |
| CG | #(CGA) | #(CGC) | #(CGU) | #(CGG) |
| UG | #(UGA) | #(UGC) | #(UGU) | #(UGG) |

^*^Four strands; A: Adenine; C: Cytosine; U: Uracil; G: Guanine.

Table ST4. Co-occurrence matrix **L** containing the pattern of nucleobases^*^ in the miRNA sequence.

|  | A | C | U | G |
| --- | --- | --- | --- | --- |
| CA | #(CAA) | #(CAC) | #(CAU) | #(CAG) |
| UA | #(UAA) | #(UAC) | #(UAU) | #(UAG) |
| GA | #(GAA) | #(GAC) | #(GAU) | #(GAG) |
| UC | #(UCA) | #(UCC) | #(UCU) | #(UCG) |
| GC | #(GCA) | #(GCC) | #(GCU) | #(GCG) |
| GU | #(GUA) | #(GUC) | #(GUU) | #(GUG) |

^*^Four strands; A: Adenine; C: Cytosine; U: Uracil; G: Guanine.

Table ST5. Co-occurrence matrix **M** containing the pattern of nucleobases^*^ in the miRNA sequence.

|  | A | C | U | G |
| --- | --- | --- | --- | --- |
| ACU | #(ACUA) | #(ACUC) | #(ACUU) | #(ACUG) |
| ACG | #(ACGA) | #(ACGC) | #(ACGU) | #(ACGG) |
| AUG | #(AUGA) | #(AUGC) | #(AUGU) | #(AUGG) |
| CUG | #(CUGA) | #(CUGC) | #(CUGU) | #(CUGG) |

^*^Four strands; A: Adenine; C: Cytosine; U: Uracil; G: Guanine.

Table ST6. Co-occurrence matrix **N** containing the pattern of nucleobases^*^ in the miRNA sequence.

|  | A | C | U | G |
| --- | --- | --- | --- | --- |
| CAU | #(CAUA) | #(CAUC) | #(CAUU) | #(CAUG) |
| CAG | #(CAGA) | #(CAGC) | #(CAGU) | #(CAGG) |
| UAG | #(UAGA) | #(UAGC) | #(UAGU) | #(UAGG) |
| UCG | #(UCGA) | #(UCGC) | #(UCGU) | #(UCGG) |

^*^Four strands; A: Adenine; C: Cytosine; U: Uracil; G: Guanine.

Table ST7. Co-occurrence matrix **O** containing the pattern of nucleobases^*^ in the miRNA sequence.

|  | A | C | U | G |
| --- | --- | --- | --- | --- |
| AUC | #(AUCA) | #(AUCC) | #(AUCU) | #(AUCG) |
| AGC | #(AGCA) | #(AGCC) | #(AGCU) | #(AGCG) |
| AGU | #(AGUA) | #(AGUC) | #(AGUU) | #(AGUG) |
| CGU | #(CGUA) | #(CGUC) | #(CGUU) | #(CGUG) |

^*^Four strands; A: Adenine; C: Cytosine; U: Uracil; G: Guanine.

Table ST8. Co-occurrence matrix **P** containing the patterns of nucleobases^*^ in the miRNA sequence.

|  | A | C | U | G |
| --- | --- | --- | --- | --- |
| UCA | #(UCAA) | #(UCAC) | #(UCAU) | #(UCAG) |
| GCA | #(GCAA) | #(GCAC) | #(GCAU) | #(GCAG) |
| GUA | #(GUAA) | #(GUAC) | #(GUAU) | #(GUAG) |
| GUC | #(GUCA) | #(GUCC) | #(GUCU) | #(GUCG) |

^*^Four strands; A: Adenine; C: Cytosine; U: Uracil; G: Guanine.

Table  [ST9](#Table5). Hyperparameter Table for DL models using K10 protocol.

| **Model Type** | **Optr*** | **CVP** | **LR** | **Epochs** |
| --- | --- | --- | --- | --- |
| EDL1: BiLSTM-BiGRU ⊕ LSTM-GRU | Adagrad | K10 | 0.2 | 50 |
| EDL2: BiLSTM-BiGRU ⊕ BiRNN-RNN | Adagrad | K10 | 0.25 | 50 |
| EDL3: BiGRU-GRU ⊕ LSTM-CNN | SGD | K10 | 0.3 | 65 |
| EDL4: BiRNN-CNN ⊕ GRU-CNN | SGD | K10 | 0.3 | 65 |
| EDL5: BiLSTM-LSTM ⊕ RNN-CNN | SGD | K10 | 0.2 | 50 |
| EDL6: BiLSTM-CNN ⊕ BiGRU-CNN | SGD | K10 | 0.3 | 50 |

*Optr: Optimizer; CVP: Cross-Validation partition; LR: Learning Rate; SGD: Stochastic Gradient Descent, K10: K10: 90%:10%.

**2. Figures**


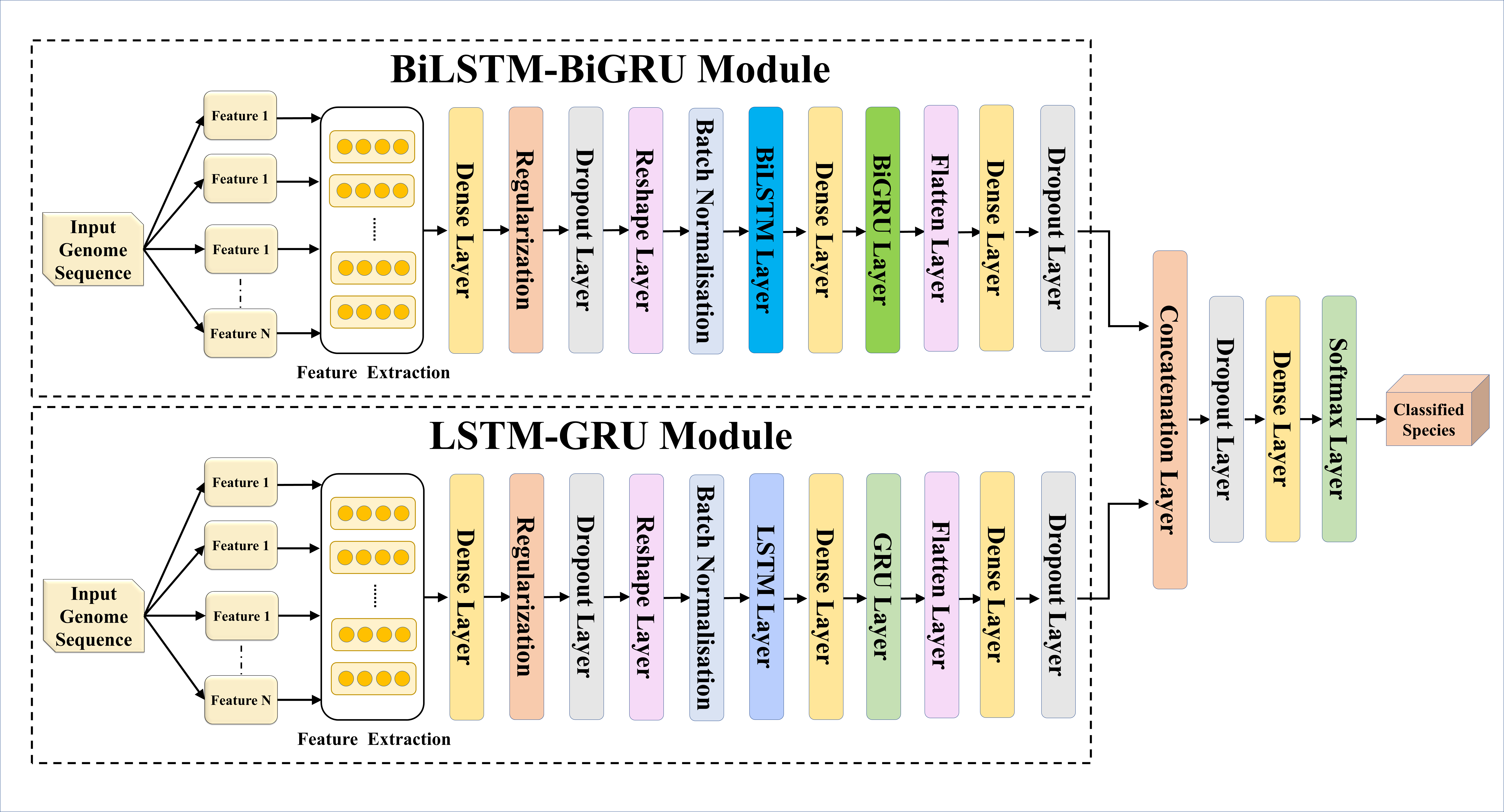


Figure F1. The architecture of the ensemble model: [BiLSTM-BiGRU ⊕ LSTM-GRU] (EDL1).


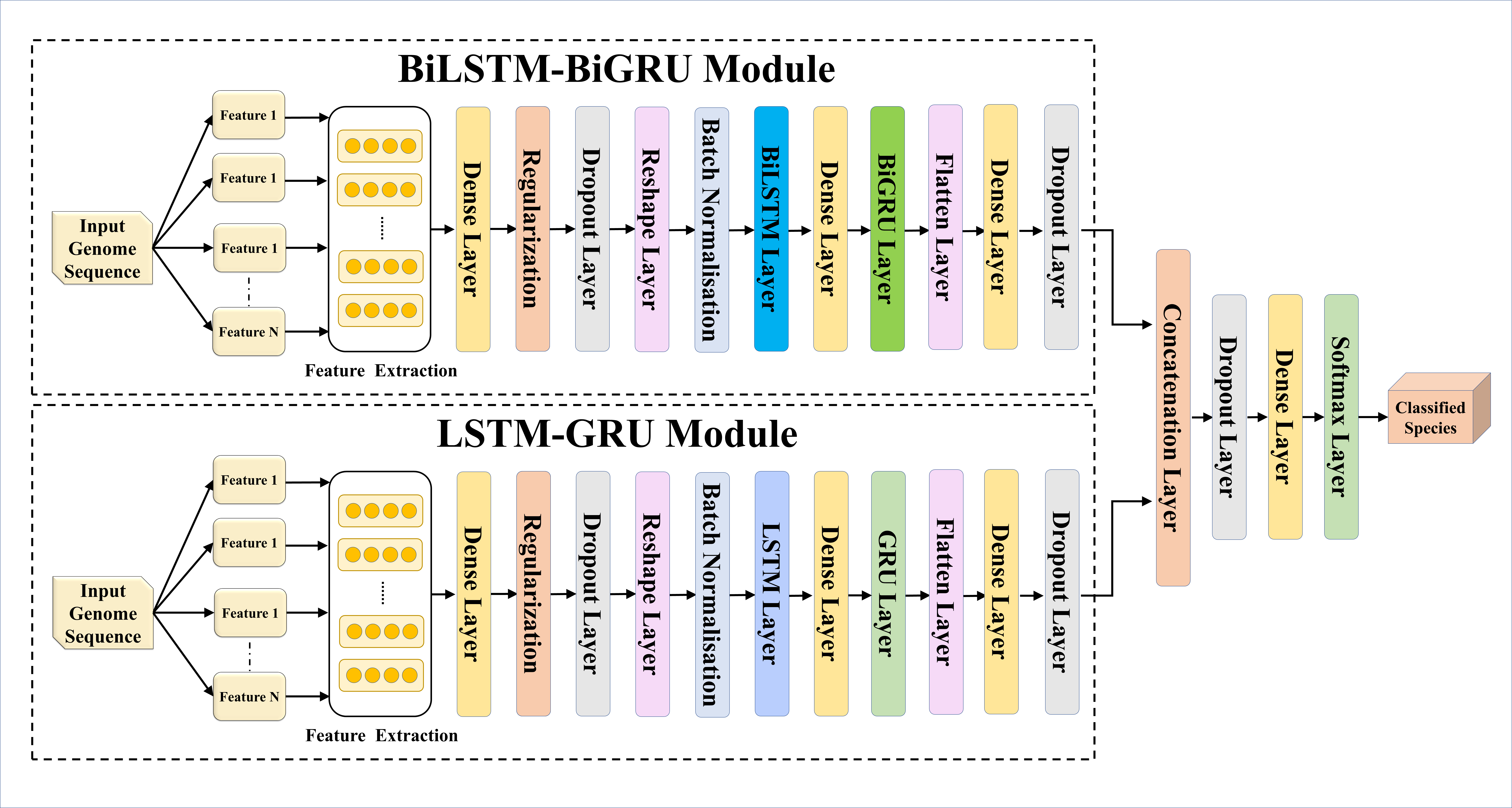


Figure F2. The architecture of the ensemble model: [BiLSTM-BiGRU ⊕ BiRNN-RNN] (EDL2).


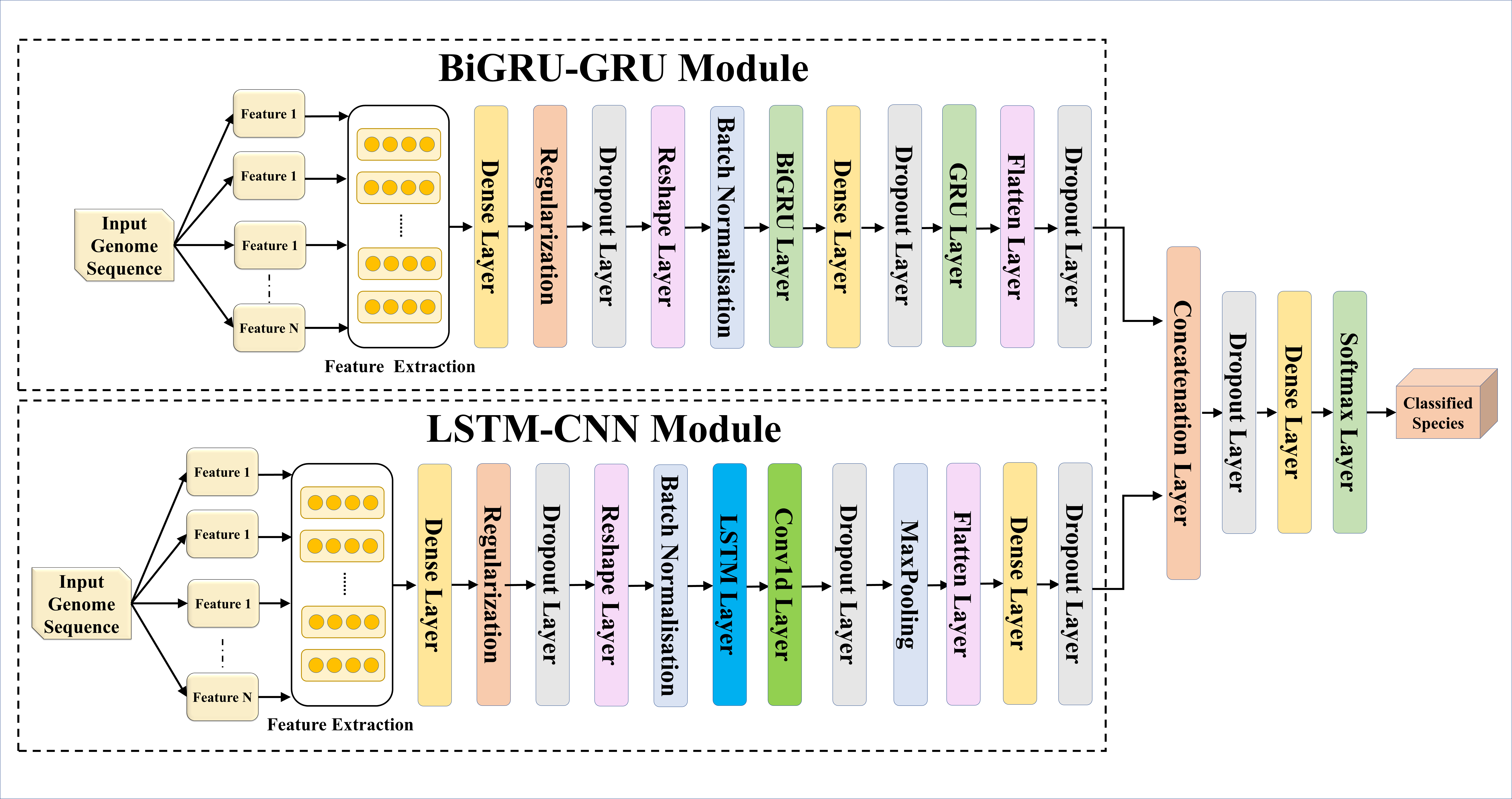


Figure F3. The architecture of the ensemble model: [BiGRU-GRU ⊕ LSTM-CNN] (EDL3).


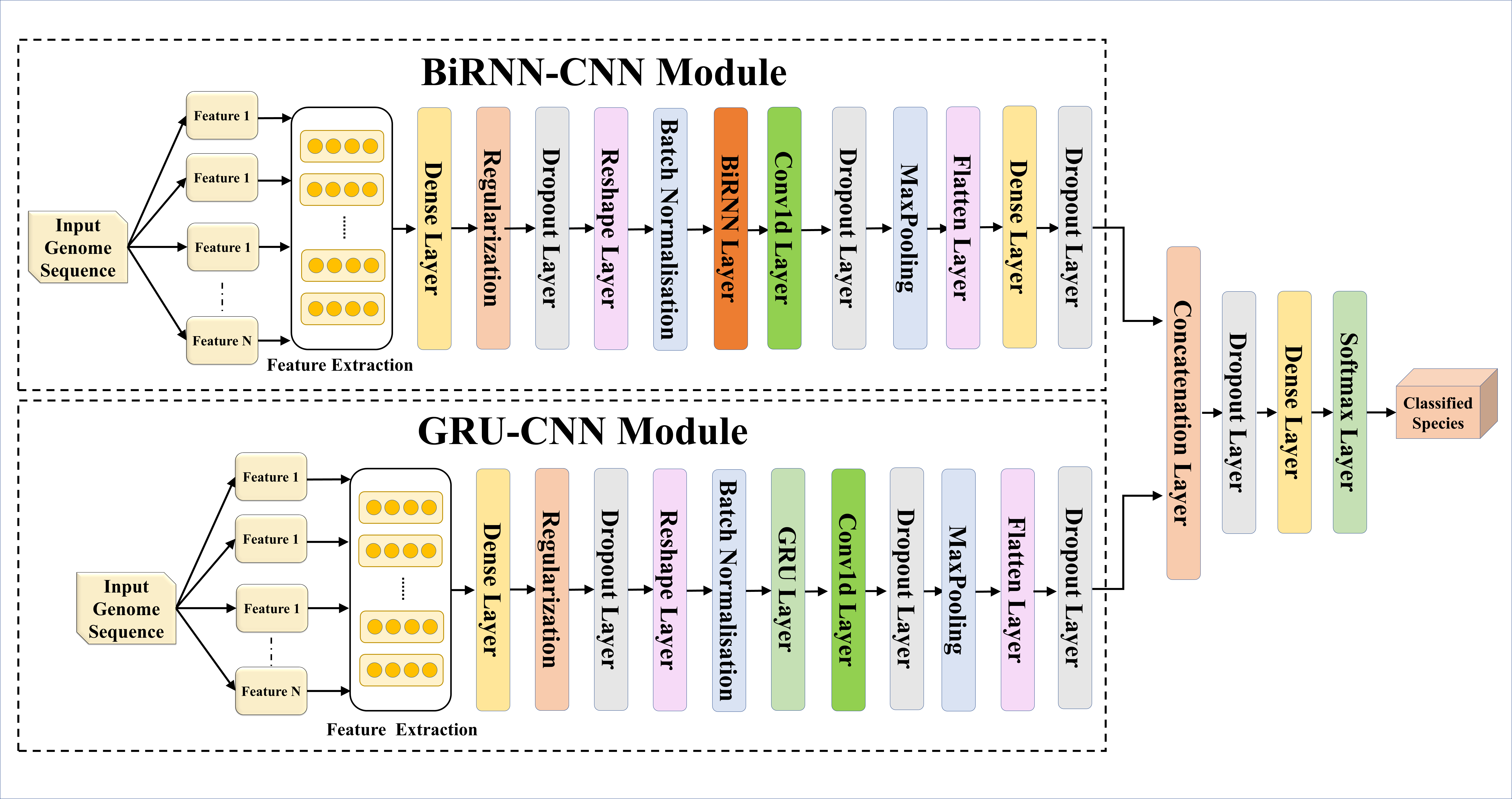


Figure F4. The architecture of the ensemble model: [BiRNN-CNN ⊕ GRU-CNN] (EDL4).


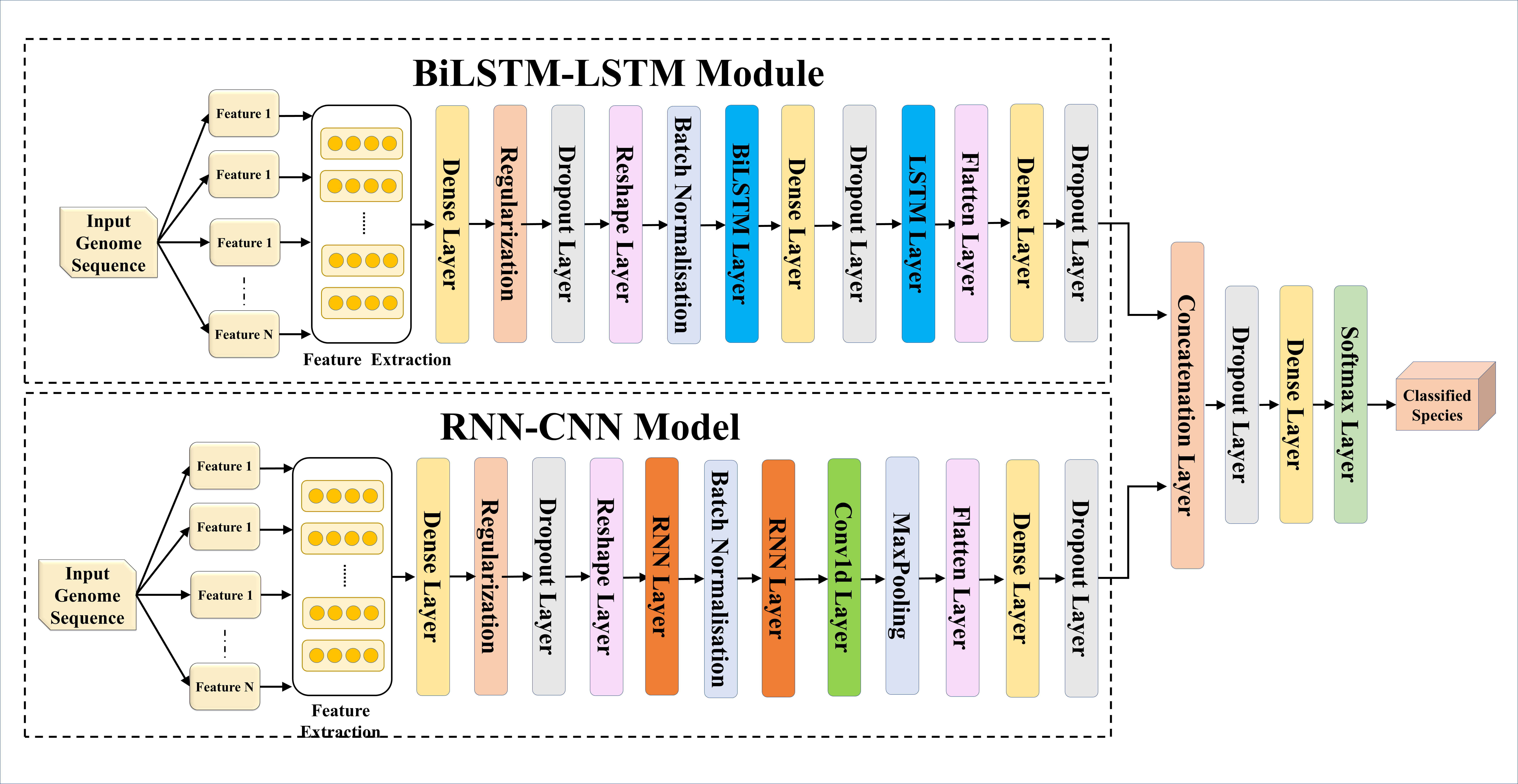


Figure F5. The architecture of the ensemble model: [BiLSTM-LSTM ⊕ RNN-CNN] (EDL5).


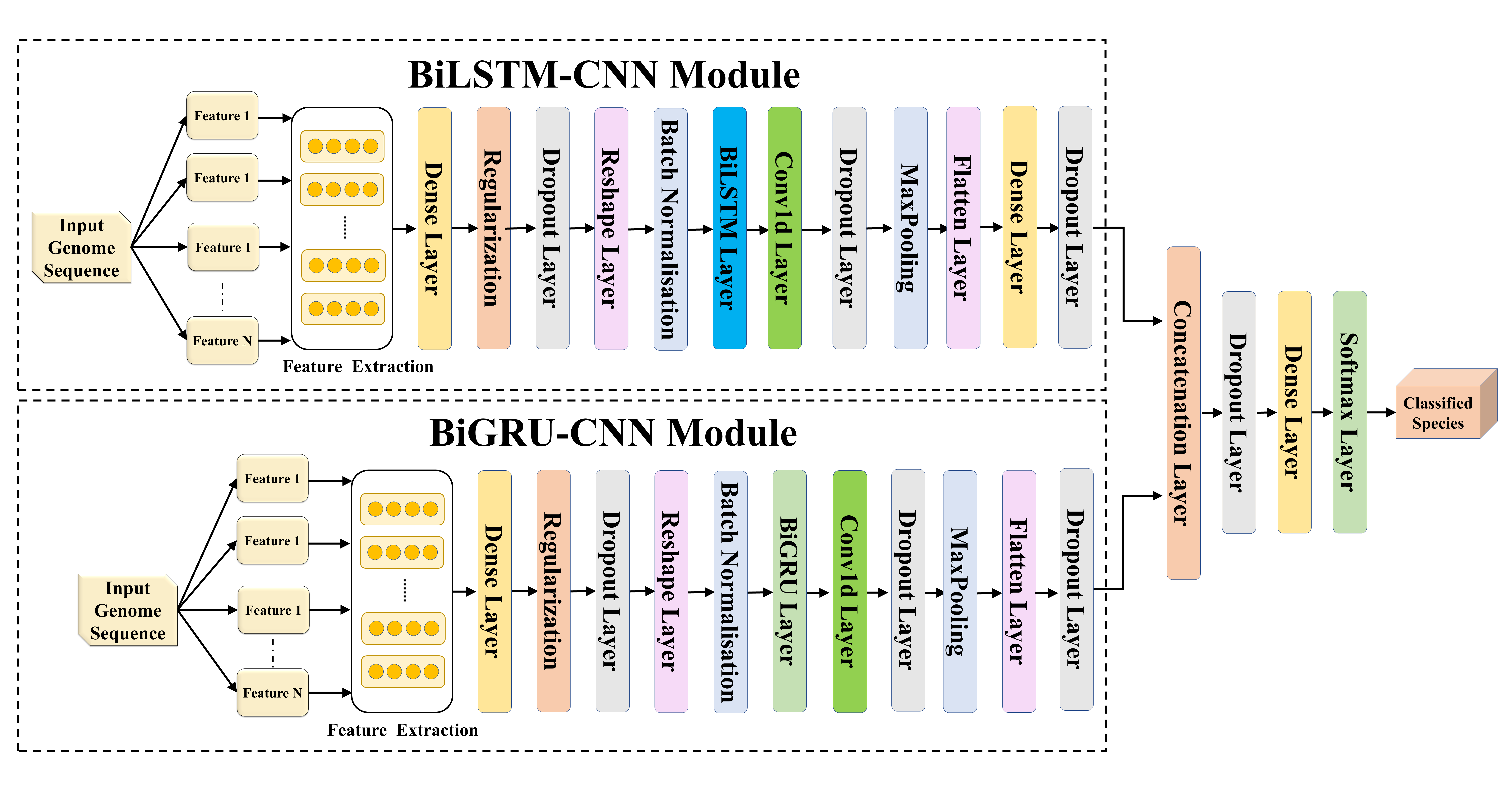


Figure F6. The architecture of the ensemble model: [BiLSTM-CNN ⊕ BiGRU-CNN] (EDL6).
